# Advances in nanomedicines for lymphatic imaging and therapy

**DOI:** 10.1186/s12951-023-02022-x

**Published:** 2023-08-24

**Authors:** Pan He, Haitian Tang, Yating Zheng, Yongfu Xiong, Hongwei Cheng, Jingdong Li, Yang Zhang, Gang Liu

**Affiliations:** 1 Department of Hepatobiliary Surgery, Academician (Expert) Workstation, Affiliated Hospital of North Sichuan Medical College, Nanchong, 637600 China; 2 State Key Laboratory of Vaccines for Infectious Diseases, Center for Molecular Imaging and Translational Medicine, Xiang An Biomedicine Laboratory, National Innovation Platform for Industry-Education Integration in Vaccine Research, School of Public Health, Xiamen University, Xiamen, 361002 China

**Keywords:** Lymph node, Nanomedicine, Specific delivery, Diagnosis, Therapy

## Abstract

Lymph nodes play a pivotal role in tumor progression as key components of the lymphatic system. However, the unique physiological structure of lymph nodes has traditionally constrained the drug delivery efficiency. Excitingly, nanomedicines have shown tremendous advantages in lymph node-specific delivery, enabling distinct recognition and diagnosis of lymph nodes, and hence laying the foundation for efficient tumor therapies. In this review, we comprehensively discuss the key factors affecting the specific enrichment of nanomedicines in lymph nodes, and systematically summarize nanomedicines for precise lymph node drug delivery and therapeutic application, including the lymphatic diagnosis and treatment nanodrugs and lymph node specific imaging and identification system. Notably, we delve into the critical challenges and considerations currently facing lymphatic nanomedicines, and futher propose effective strategies to address these issues. This review encapsulates recent findings, clinical applications, and future prospects for designing effective nanocarriers for lymphatic system targeting, with potential implications for improving cancer treatment strategies.

## Introduction

Cancer is a significant health threat with high incidence and mortality rates, and most cancer-related deaths are attributed to metastasis. Only a fraction of metastatic patients, approximately one-fifth, achieve a long-term survival time of over five years [1]. Metastasis occurs when tumor cells from the primary site spread and colonize organs throughout the body, resulting in organ dysfunction and failure, as observed in prostate cancer, lung cancer, pancreatic cancer, and other malignancies [2, 3]. Common routes of cancer metastasis include hematogenous, implantation, and lymph node metastasis. Although a large amount of research focuses on the hematogenous route of cancer metastasis, the majority of metastases in epithelial tumors originate from lymph nodes spreading through the lymphatic system [4]. Clinically, lymph nodes surrounding the cancer lesions are often examined to verify whether metastasis has occurred, aiding in tumor staging and determination of treatment plans. Additionally, research indicates that lymph node metastasis not only plays an important role in the distant metastasis process but also has a significant impact on local recurrence [5]. The treatment plan and prognosis of a patient are directly related to the involvement and effective intervention of the lymph nodes. Furthermore, as an essential component of the immune system, lymph nodes contain a rich population of immune cells, providing a platform for effective immunotherapy [6]. Thus, harnessing the characteristics of the lymphatic system for accurate identification of lymph nodes and targeted drug delivery is paramount for improving cancer diagnosis, staging, and therapeutic efficacy. Developing drug delivery systems based on the lymphatic system is an effective approach to improving cancer treatment. With the rapid advancement of drug preparation technology, novel drug delivery systems exhibit unique features, among which nanomedicine based on nanotechnology holds great promise and garners significant attention.

Nanomedicine exhibits excellent tumor delivery efficiency due to its high penetration and retention effects in solid tumors [7], which has significant advantages in cancer recognition, diagnosis, and treatment of metastasis [8]. These advantages include: (1) Nanocarriers possess more effective and controllable biological activity and imaging performance, and can improve the solubility, bioavailability, and stability of the delivered drugs through chemical functionalization, hydrophilic-hydrophobic interactions, and electrostatic interactions [9]; (2) Nanomedicine can improve drug pharmacokinetics and tissue distribution by extending circulation time or preventing rapid clearance by the reticuloendothelial system [10]; (3) The high surface area of nanomedicine is conducive to more surface modification, and efficient drug surface functionalization can achieve personalized diagnosis and treatment applications; when targeted ligands are surface-modified, targeted tissue or cell enrichment and bioavailability of target lesions can be improved through receptor-ligand interactions [11]. Based on these advantages of nanomedicine, the US Food and Drug Administration (FDA) and the European Medicines Agency (EMA) have approved a variety of nanomedicine for the diagnosis, treatment, and imaging recognition of cancer and metastasis, such as nanocarbon and abraxane, et al. [12, 13], which has a promising future for improving the dilemma of cancer treatment.

**Fig. 1:**
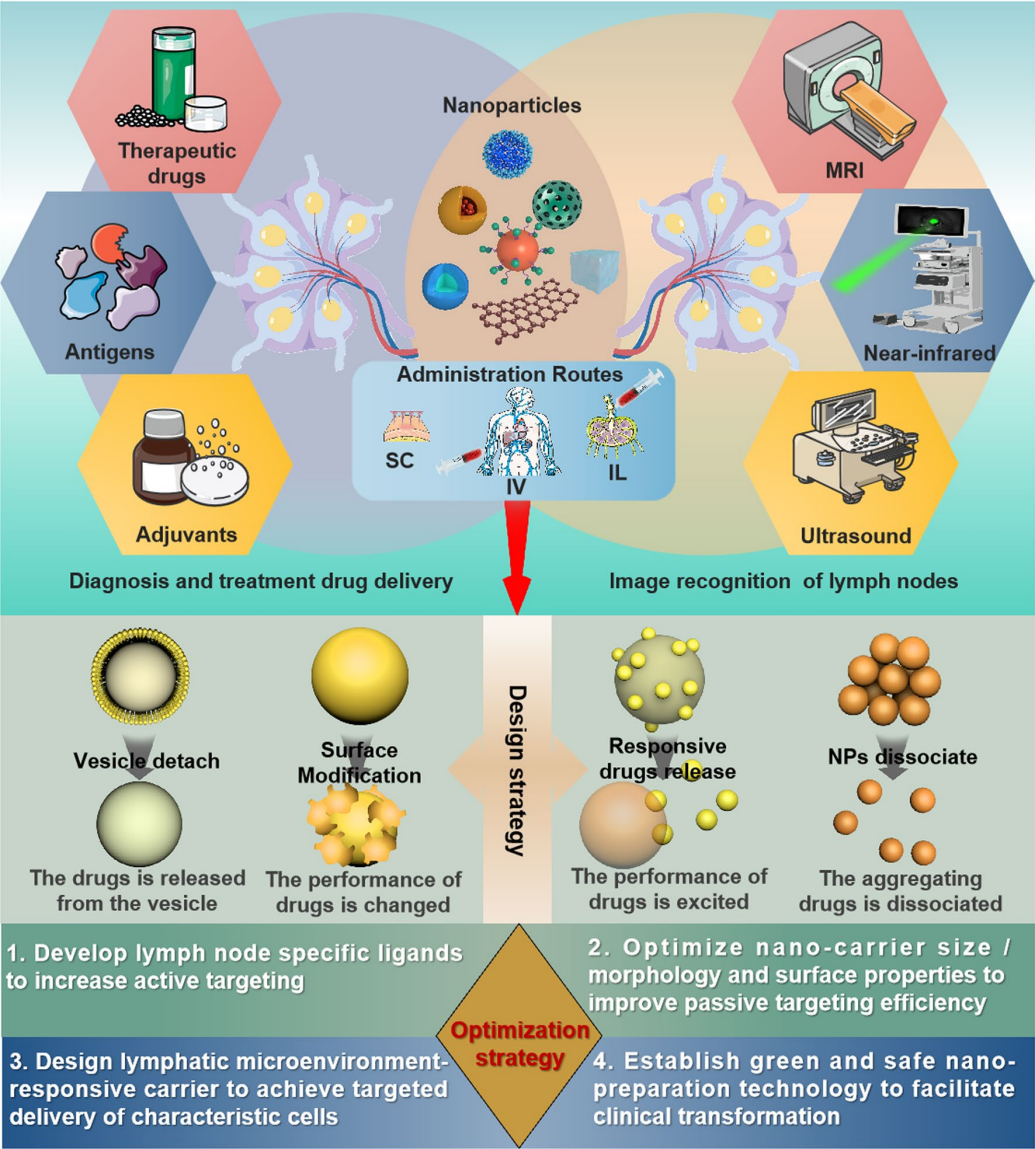
A schematic represents the application of lymph node-specific nanomedicine (upper panel), design strategies (middle panel) and optimization strategies (below panel). Upper panel shows the schematic of the main application of lymph node-specific nanoparticles, including lymph node diagnosis and drug delivery and enhanced image recognition. And the mode of administration of subcutaneous injection, intravenous injection and intralymphatic injection. Middle panel illustrates the lymph node specific nanomedicine design strategies including vesicle detach, surface modification, responsive drug release, and NPs dissociate. Below panel illustrates the lymph node specific nanomedicine optimization strategies

Targeted intervention of the lymphatic system is an effective way to improve cancer treatment. Many studies have emphasized the potential of lymphatic-targeted nanomedicine in disease diagnosis, imaging recognition, and metastasis treatment [14, 15]. However, the complex anatomical structure and physiological function of the lymphatic system present challenges in designing and developing effective lymphatic-targeted nanocarriers for drug delivery, limiting clinical translation [16]. Based on the reported methods and design criteria of lymphatic nanomedicine delivery systems [17], this review focuses on the materials used for targeted delivery of nanocarriers for diagnosis, treatment, and imaging recognition of the lymphatic system (Fig. 1). Firstly, the composition, physiological function, and factors affecting lymphatic nanomedicine delivery of the lymphatic system are introduced. Then, the design and application of lymphatic-targeted nanocarriers are discussed, with a particular focus on the design strategies, physicochemical properties, and current clinical applications of delivery carriers in diagnosis, treatment, and imaging recognition. Finally, we discuss the complexity, potential pitfalls, and opportunities of local drug delivery techniques in lymphatic nanomedicine delivery systems.

## The composition and physiological functions of the lymphatic system

**Fig. 2:**
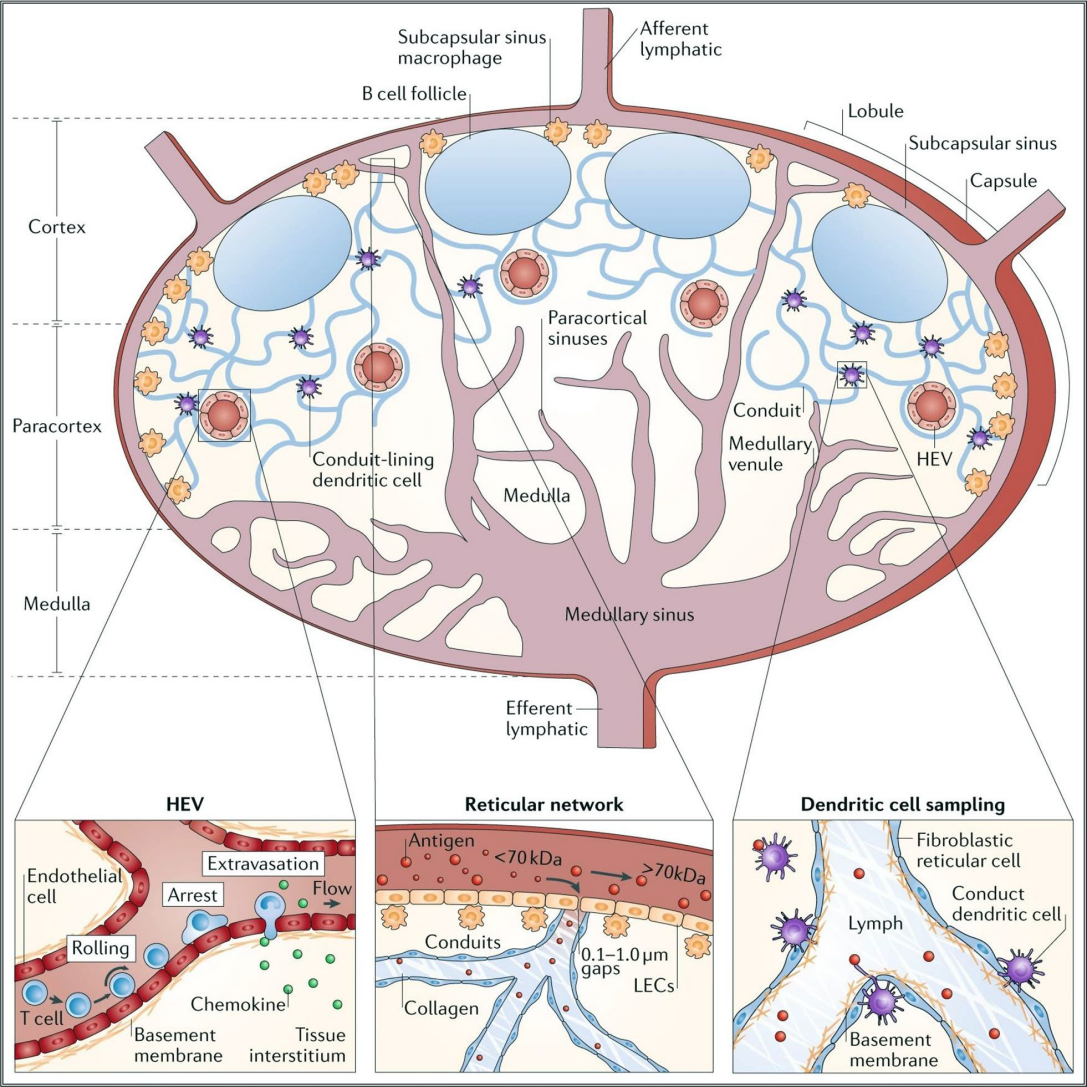
Structure and physiology of lymph nodes. A cross section of a lymph node is shown. The architecture of the lymph node can be divided into distinct areas: fluid-filled lumen structures (lymphatics, high endothelial venules (HEVs), capillaries and sinuses), cellular locations (B cells in follicles, dendritic cells and T cells in the paracortex and macrophages in the subcapsular sinus and medulla) and structural units (cortex, paracortex and medulla). Lymphocyte extravasation occurs in the HEVs. The distribution of antigens within the reticular structure is regulated by haemodynamic size and molecular weight by the capsule and conduit. Circulating lymphocytes enter through the vasculature and exit through the efferent lymphatics [22]. Copyright © 2019 Springer Nature

The lymphatic system, comprising of primitive lymphatic vessels formed by lymphatic endothelial cells, serves as a crucial conduit for the body. The capillary lymphatic vessels exhibit incomplete intercellular junctions, thereby enabling the transport of interstitial fluid via a process influenced by the mechanical expansion instigated by skeletal muscle movements and lymphatic drainage. This permits a unidirectional transit of body fluids, solutes, and cells from the peripheral tissues towards lymph nodes [18, 19]. The fluid assimilated into the lymphatic system, referred to as lymph, subsequently merges into larger collecting lymphatic vessels. The propulsion of lymphatic fluid in the lymphatic system comes from the active pumping motion of surrounding smooth muscle cells, and is directed by one-way valve structures that restrict backflow and guide the fluid away from the tissue drainage site and towards the direction of flow [20, 21]. Lymph nodes are distributed along the lymphatic network, and their structure and physiology are shown in Fig. 2. Before the lymph ultimately returns to the subclavian vein, immune cells sample and filter antigens carried by the lymph [22]. It has been reported that most of the proteins carried by lymph come from the filtering at the first draining lymph node [23].

The special structure of lymph nodes not only guides the concentration and interaction of antigens, antigen presenting cells, and lymphocytes, but also affects the biological distribution of cancer cells, pathogens, or nanoparticles in the lymph. Specifically, lymph enters the lymph node through the afferent lymphatic vessels in the subcapsular sinus (SCS). The SCS is composed of a single layer of LECs interspersed with macrophages and dendritic cells that collect lymph, and this single-layer structure can impede lymph from entering the lymph node parenchyma and guide lymph to diffuse through the cortical sinuses [24, 25]. LECs are also arranged in the SCS that connects to the medullary, transverse, and cortical sinus structures [26], and finally exits the lymph node through the efferent lymphatic vessels [27]. Nonetheless, a canal system composed of collagen fiber networks in the above process, enabling lymph and solutes within the SCS to infiltrate deeper into the lymph node parenchyma, such as most of the resident white blood cells [28, 29]. In addition, the lymphatic system is an important carrier for immune cells and proliferating cancer cells and plays an important role in the occurrence, development, and metastasis of cancer. As the main site of cancer metastasis, fully utilizing the unique nanostructure biological distribution performance of lymph nodes for effective intervention can be an effective approach for cancer treatment.

## Factors affecting the specific delivery of nanomedicine to the lymphatic system

The lymphatic system has also been increasingly recognized as a valuable pathway for the targeted delivery of nanomedicines. This targeted approach can potentially enhance the therapeutic efficacy of drugs while minimizing systemic side effects, especially for diseases primarily affecting the lymphatic system such as lymphomas and lymphatic metastasis of solid tumors. The specific enrichment of nanomedicines within the lymph nodes, however, is contingent on a multitude of factors including nanoparticle characteristics (size, shape, surface charge, and composition), drug properties, as well as physiological conditions. Importantly, nanoparticles must be designed and engineered in a manner that allows them to exploit the unique anatomical and physiological features of the lymphatic system for enhanced lymphatic uptake, retention, and subsequent lymph node targeting. Among the physicochemical properties, the nanoparticle size, shape, and surface properties of nanomedicines are the most important factors. We will briefly introduce these factors below.

### Nanoparticle size

The size determine of particles has a significant impact on their absorption and retention effects in the lymphatic system [30]. For example, larger particles can be absorbed into the lymphatic system due to barrier effects that limit their absorption by the portal vein system. While nanoparticles with smaller diameters are typically more efficient in entering the lymphatic vessels and reaching the lymph nodes. This is mainly due to the unique structure of lymphatic capillaries, which are designed to take up interstitial fluid and small particles from the surrounding tissues. The size of nanoparticles also influences their rate of transport within the lymphatic system [31]. Smaller nanoparticles (below 10 nm) are rapidly transported to the bloodstream, leading to less accumulation in the lymph nodes. Intermediate-sized nanoparticles (10–100 nm) exhibit higher lymph node accumulation because they can effectively enter the lymphatic capillaries and are transported at a slower pace, allowing for their retention in the lymph nodes. However, nanoparticles larger than 100 nm might have limited access to lymphatic capillaries, and hence, less efficiently reach the lymph nodes.

Another important parameter that affects the absorption of nanomedicines by the lymphatic system is the molecular weight. The higher the molecular weight, the stronger the absorption performance of the lymphatic system. Because the exchange capacity of capillaries becomes narrow, entering the lymphatic system becomes a way for drugs with higher molecular weight [32]. Studies have shown that when the molecular weight of the drug is greater than 16,000 D, the absorption of the lymphatic system will increase significantly, especially for colloidal materials, which can achieve active targeting due to their high molecular weight [33].

Therefore, nanoparticle size is a crucial parameter in designing efficient drug delivery systems targeting the lymphatic system. Properly sized nanoparticles can achieve optimal lymphatic uptake, transport, and lymph node accumulation, contributing to improved therapeutic outcomes.

### Nanoparticle shape

The shape of nanoparticles plays a critical role in their enrichment in lymph nodes, largely by affecting their transportation, biodistribution, cellular uptake, and clearance. Nanoparticles of various shapes, such as spherical, rod-like, discoidal, or worm-like, interact differently with biological systems. In terms of lymphatic uptake and transport, some studies have indicated that rod-shaped nanoparticles can have higher lymphatic uptake compared to spherical ones due to the elongated aspect ratio [34, 35]. This shape can mimic the natural aspect ratio of viruses and proteins that the lymphatic system is adapted to transport, enhancing their movement through the lymphatic system to the lymph nodes. The shape of nanoparticles can also influence their interaction with immune cells in the lymph nodes [36]. For instance, non-spherical nanoparticles may have higher interactions with dendritic cells, macrophages, and other antigen-presenting cells (APCs) due to their increased surface area, leading to improved immunostimulation. Therefore, the shape of nanoparticles must be carefully considered when designing targeted drug delivery systems for the lymphatic system.

### Surface properties of nanoparticle

In terms of surface properties, the factors that affect the absorption of nanomedicine by the lymphatic system are mainly surface charge and hydrophilic-lipophilic balance. For example, when using liposomes as drug delivery carriers, negatively charged liposomes are more favorable for lymphatic absorption than positively charged liposomes. Patel found through labeling liposomes with 125 iodine that the absorption order of lymphatic system was negative > positive > neutral. Moreover, it has been found that compared with positively charged liposomes, negatively charged liposomes have longer retention time in the lymphatic system [37]. Therefore, by changing the surface charge of nanodrugs, the uptake of cells and the transport and bioavailability of nanodrugs by lymphatic system can be enhanced. In order to improve the lymphatic absorption performance of nanodrugs, the hydrophilic-lipophilic balance of the surface properties of nanodrugs should also be considered. An appropriate balance between hydrophilicity and lipophilicity can achieve optimal lymphatic absorption. It is generally believed that when the distribution coefficient or logarithmic *P* value is greater than 4.7, it means that the lymphatic affinity is about 50,000 times that of the blood inlet and it is conducive to effective dissolution in triglycerides and lymphatic absorption [38].

### Administration route

Every route of drug administration possesses its inherent characteristics, which can significantly alter the biological distribution and metabolic behavior of a drug within the body, thereby significantly affecting the efficiency of drug accumulation at the target site. In general, for nanodrugs to achieve lymph node targeting, they first need to efficiently enter the lymphatic system [39]. Hence, the degree of contact between the drug administration method and the lymphatic system is an important factor. The lymphatic system is a crucial site for immune responses, so nanodrugs are prone to come into contact with the immune environment, which enhances their capture by immune cells, thereby improving the efficiency of their entrance into the lymph nodes. In addition, the biological barriers faced by different routes of administration vary, which can severely affect the bioavailability of the drug [40]. Typically, routes of administration include oral, intravenous, nasal, subcutaneous, and lesion injection.

Subcutaneous administration is the most common and effective method for achieving lymph node targeting [41, 42]. This is because subcutaneous administration allows drugs to directly contact the skin environment rich in immune cells. These immune cells capture the nano-drugs and transport them to the lymph nodes via lymph vessels. Furthermore, subcutaneous administration, as a local injection, can significantly improve the retention of the drug, thereby enhancing its efficacy. For instance, some researchers have loaded nanoparticles onto microneedle patches, which when applied directly to the skin, can facilitate dermal administration [43]. Microneedles have sustained-release capabilities, thus enabling a slow and continuous release of the nano-drugs. These are then captured by the dendritic cells in the skin and transported to the lymph nodes, facilitating the specific activation of T cells and achieving long-term immunotherapeutic effects. Intravenous injection allows drugs to enter the systemic circulation, thereby reaching all parts of the body, including the lymph nodes. However, nanodrugs administered intravenously may be rapidly cleared by the kidneys or liver. Therefore, the efficiency of lymph node targeting by this method is usually low, as the drug first needs to enter the interstitial fluid from the blood vessels before entering the lymphatic system [44]. However, studies have shown that specially designed nanodrugs can significantly avoid clearance by the kidneys and liver while effectively targeting the lymph nodes. Intranasal administration is another non-invasive route of administration that can target nasal-related lymphoid tissues. However, most drugs may be cleared and swallowed by mucociliary movement, limiting the quantity of drugs entering the lymphatic system [45]. Direct injection into the lymph vessels or lymph nodes can directly administer drugs into these structures, thereby achieving extremely high lymph node targeting efficiency. However, this method requires high technical skills and is highly invasive [46]. While orally administered drugs need to undergo stomach acid breakdown, intestinal absorption, and first-pass metabolism in the liver, all of which can reduce the bioavailability of the drug and affect its ability to reach the lymph nodes, thus, it is generally few used for lymph node administration [47].

In summary, the enrichment of nanoparticles in lymph nodes is a multifactorial process influenced by various aspects of nanoparticle design. Three critical parameters, size, shape, and surface properties, govern the behavior of these nanoparticles within the biological environment and their subsequent accumulation within the lymphatic system. By optimizing these parameters, nanoparticles can be designed to have enhanced lymphatic uptake and accumulation, providing improved efficacy for the delivery of drugs, vaccines, and diagnostic agents to the lymphatic system. As we further understand the impact of these factors on nanoparticle behavior in biological systems, we can better exploit the unique properties of nanoparticles for effective lymphatic targeting.

## Types of nanocarriers for lymphatic system drug delivery

Nanocarrier-based drug delivery systems have been widely used to improve drug targeting, enhance bioavailability, and prolong circulation time. When combined with lymph node-based therapies and imaging recognition, nanocarriers can effectively enhance cancer immunotherapy and surgical resection. In this section, we will mainly summarize the nanocarrier materials used for lymphatic system drug delivery from two aspects: disease diagnosis and treatment, and lymph node imaging recognition and clearance. We will also review the latest research progress and provide insights for designing better targeted nanocarriers for the lymphatic system in the future.

## Lymphatic diagnosis and treatment nanomedicines

### Liposome-based nanoparticles

**Fig. 3:**
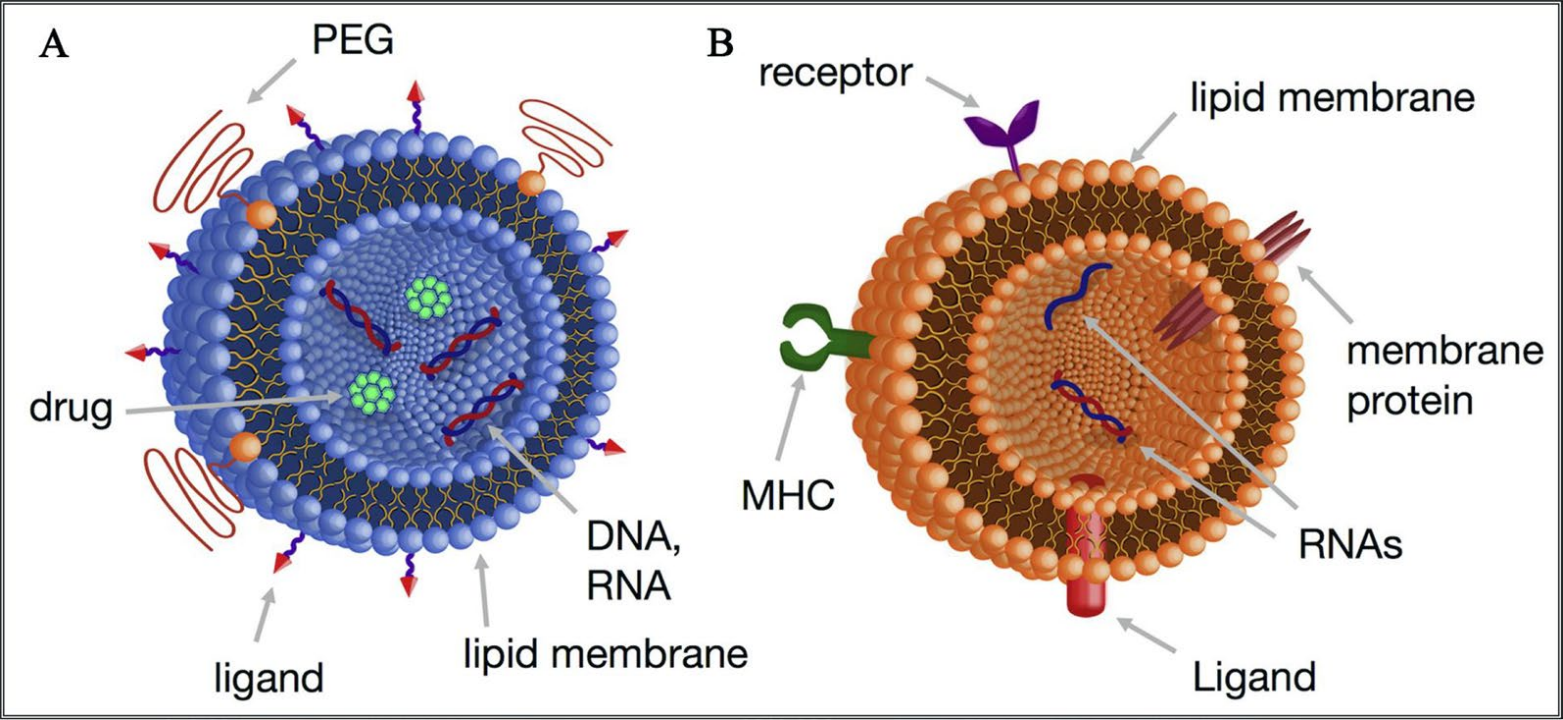
Design of cationic liposome nanomaterials for lymphatic targeting based on polyethylene glycol surface modification and functionalization. **A** An example of a lipid nanoparticle composed of phospholipids, targeting ligands, PEGylated lipids, drugs, and nucleic acids. **B** An example of an extracellular vesicle (EV) containing phospholipids, receptors, proteins, nucleic acids, MHC (major histocompatibility complex) molecules, and ligands [53]. Copyright © 2018 Elsevier B.V

Liposomes, also known as phospholipid vesicles, were discovered in the 1960s and are composed of phospholipid bilayers with a hollow structure [48, 49]. As a drug delivery carrier, the phospholipid bilayer and hollow structure of liposomes can carry various drugs [50]. Although liposomes have good biocompatibility, their targeting ability is poor. To achieve ideal use, effective targeting proteins need to be integrated during the use of liposomes, or the particle size, charge, and surface properties of the liposome targeting carrier need to be improved to achieve greater absorption and accumulation in the lymphatic system [51, 52]. As shown in Fig. 3A, surface modification using polyethylene glycol (PEG)-lipids enables the development of pH-sensitive cationic lipids and the design of multifunctional lipid nanoparticles. This strategy has significantly enhanced the performance of lipid nanoparticles. PEGylated lipid nanoparticles, known as Doxil^®^, have been approved as anticancer drugs, while pH-sensitive cationic lipid nanoparticles are currently undergoing clinical trials. Furthermore, extracellular vesicles composed of proteins, mRNA, miRNA, and DNA, and prepared by embedding membrane proteins, MHC molecules, and integrins into phospholipid membranes, provide an ideal carrier for the delivery of nuclear medicine (Fig. 3B). In other words, extracellular vesicles can be considered as naturally occurring drug delivery vehicles within the human body. In terms of disease diagnosis and treatment, liposome-based nanoparticle carriers targeting the lymphatic system are mainly used for effective delivery of vaccines to achieve efficient anti-tumor immune activation [53].

**Fig. 4:**
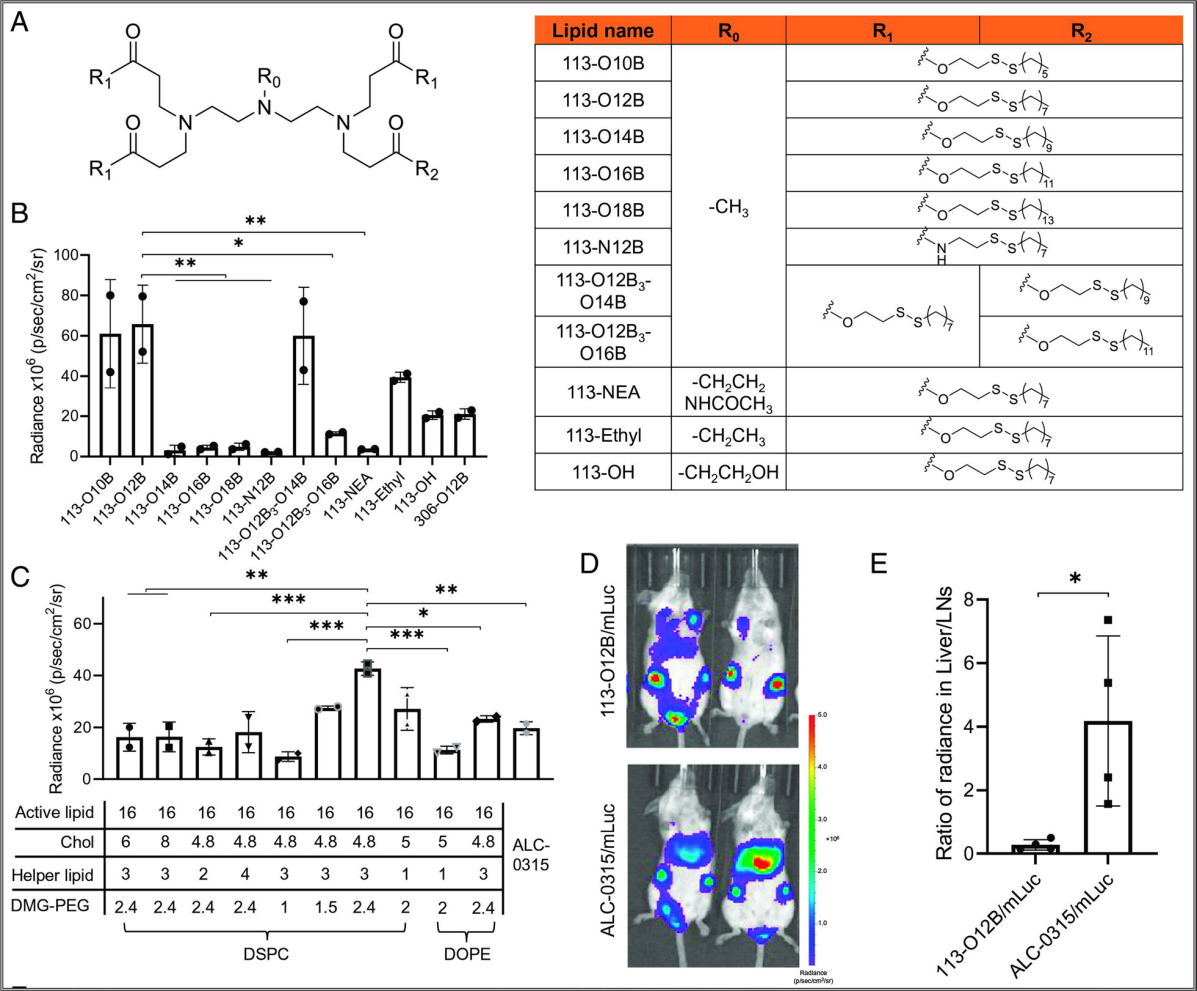
Screening and optimization of lipid nanoparticles (LNPs) with targeting ability to lymph nodes (LNs). **A** The chemical structure of lipids used in this study. **B** The bioluminescence within inguinal LNs after treatment with LNP/mLuc. **C** The bioluminescence within inguinal LNs after treatment by LNP/mLuc. **D** Representative images of bioluminescence distribution in mice treated with 113-O12B/mLuc and ALC-0315/mLuc. **E** Ratio of radiance in liver and inguinal LNs after subcutaneous injection of mLuc [58]. Copyright © 2022 National Academy of Sciences

Vaccine adjuvants can promote antigen uptake and induce immune responses at low doses for cancer treatment. Among them, adjuvants applied to protein vaccines are the most common, especially liposomes prepared from dimethyl dioctadecyl ammonium bromide (DDAB) and trehalose-6,6-dibehenate (TDB) as raw materials can be an effective vaccine adjuvant. It has been reported that small molecule monolayer liposomes composed of DDA and TDB can induce a strong CD8^+^ T cell response without the need for Toll-like receptor (TLR) agonists, effectively avoiding the potential risks brought by TLR agonists. Moreover, the use of biotin-avidin complex can further prolong the retention time of DDAB:TDB liposomes in the lymphatic system [54]. For example, a study developed a nanovaccine based on polyethylene glycol phospholipid derivatives and peptides, which showed strong tumor specificity and immunogenicity [55]. In addition to adjuvants for protein vaccines, liposomes can also be used as adjuvants for nucleic acid vaccines. Numerous studies have shown that liposomes can promote the entry of nucleic acid vaccines into the cytoplasm and prevent their enzyme degradation, with strong anti-tumor effects. For example, a cationic lipid YSK12-C4 was developed and assembled it with siRNA-containing nanoparticles to synthesize an efficient non-viral carrier, which could efficiently transfer siRNA to dendritic cell (DC) and promote gene silencing in mouse DC, enhancing tumor immune response [56]. In addition to being used as a vaccine adjuvant delivery, the new antigen-lipid nanovaccine can also enhance tumor suppression when combined with anti-PD1 antibodies or Treg inhibitory peptide P60, providing an effective synergistic treatment strategy for tumor immunotherapy [57]. Furthermore, lipid nanoparticle also exhibited a significant lymph node targeting ability [58]. A research study focuses on a lymph node-targeted mRNA vaccine based on lipid nanoparticles. This vaccine utilizes a specific delivery system to introduce mRNA encoding antigen targets into the body, leading to protein expression and stimulation of a specific immune response, thereby providing immune protection to the body. As shown in Fig. 4A, illustrates the structure of the lipid nanoparticle carrier, including the side chain, linker type, tail length, and tail composition. It also shows the influence of the chemical structure on mRNA expression within the lipid nanoparticle carrier (Fig. 4B). In this system, active lipids, cholesterol (Chol), helper lipids, and DMG-PEG all affect mRNA transfection within the lipid nanoparticle carrier (Fig. 4C). Following injection in a mouse model, both 113-O12B and ALC-0315 exhibit noticeable drainage signals, with ALC-0315 showing significantly higher expression in the liver (Fig. 4D, E). This confirms that 113-O12B possesses superior lymphatic targeting capability. Additionally, phosphatidyl serine also could be modified onto lipid nanoparticle to enhance uptake by peritoneal macrophages [59], which are then delivered to the lymphatic system to exert their immune-activating effects.

### Micelles based nanoparticles

Micelles are ordered molecular aggregates composed of amphiphilic single-layer molecules with hydrophilic and hydrophobic parts, which can take on various shapes, such as spherical, layered, and rod-shaped structures [60, 61]. During micelle formation, the hydrophilic polar groups form the outer layer, while the hydrophobic polar groups aggregate to form the core of the micelle, which can effectively carry hydrophobic drugs, improve the solubility of molecules, and prolong the circulation time of drugs in the body [62]. Micelles designed in nanodrug delivery systems are expected to achieve efficient anti-tumor therapy under low toxicity conditions. For example, micelle polymers based on methoxy-polyethylene glycol-distearoyl phosphatidylethanolamine and doxorubicin can increase the uptake of A375 cells to doxorubicin. If injected subcutaneously, the micelles absorbed by the lymphatic system can aggregate in lymph nodes and kill tumor cells within the lymph nodes [63].

A large number of T cells and dendritic cells accumulate in lymph nodes. Intervention in lymph nodes can achieve effective immunotherapy. A lot of research has shown that micelle drug delivery carriers targeted to the lymphatic system have a good impact on cellular immune function in the body [64, 65]. For example, Jewell found that the nano delivery system prepared by encapsulating Toll-like receptor-3 ligand polyinosinic-polycytidylic acid (PolyIC) in biodegradable polypropylene glycol-ethylene glycol copolymer particles had excellent sustained release effect and lymph node retention time. It can increase the accumulation of Toll-like receptor agonists in APC in lymph nodes and persistently activate DC for immunotherapy [66].

**Fig. 5:**
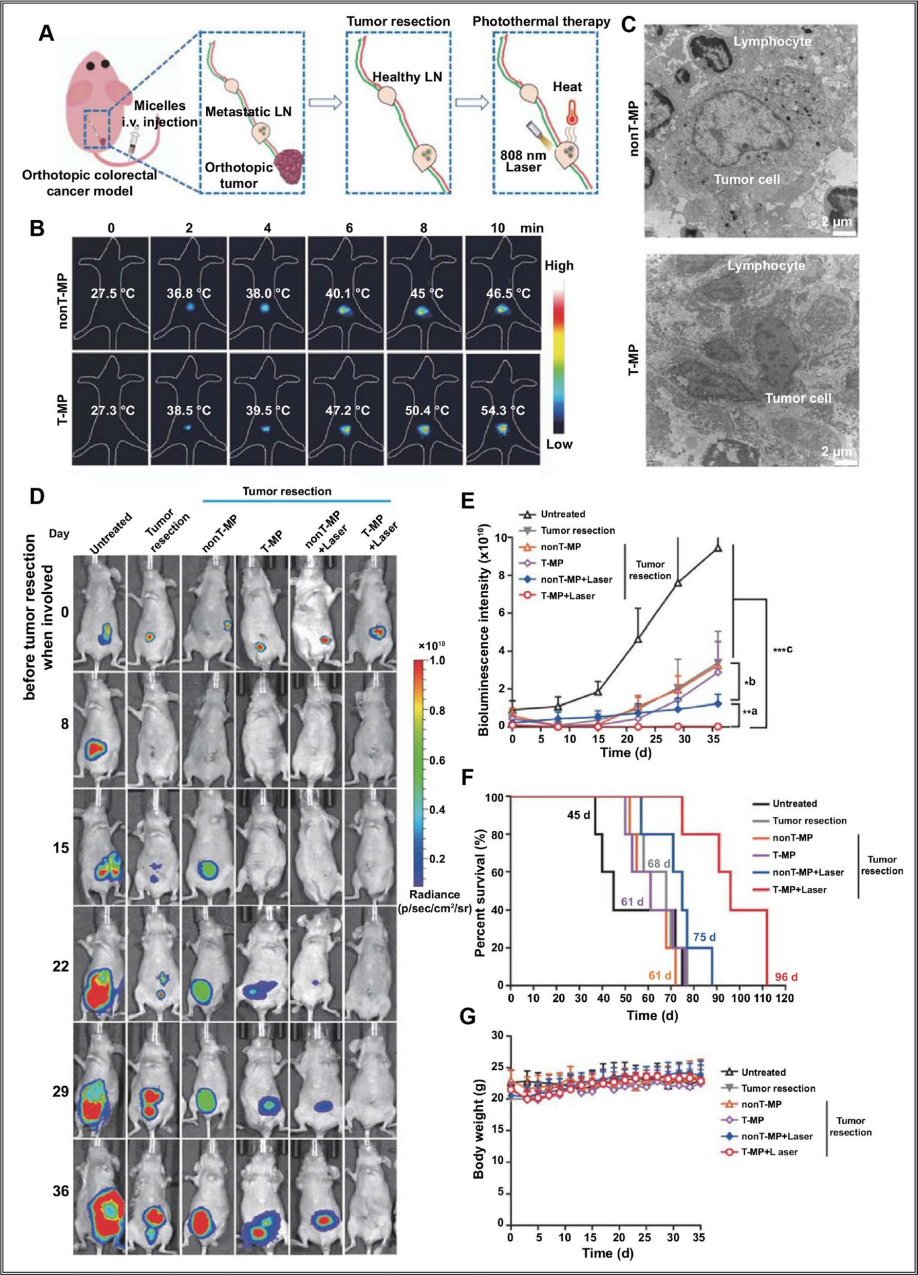
Photothermal therapy and anti-tumor evaluation of T-MP in metastatic sentinel lymph nodes in a mouse model of HER2 HT-29 colon cancer cells. **A** Illustration of the treatment of the surgical resection of orthotopic tumor and follow-up laser irradiation of the meta-static LNs. **B** Temperature increase of the metastatic LNs was recorded using Testo 890 thermal imager. **C** Representative TEM images of LN sections after photothermal therapy. **D** Representative bioluminescence imaging of the mice before tumor resection and after tumor resection and photothermal therapy. **E** Quantified bioluminescence intensity in panel **D**. **F** Survival of the mice with indicated treatments. **G** Mice body weight [69]. Copyright © 2021 OAHOST

In addition, micelles can aggregate in lymph nodes to effectively inhibit tumor lymphatic metastasis. For instance, β-benzyloxycarbonyl-L-aspartic acid-polyethylene glycol was utilized to produce paclitaxel micelles that targeted the lymph nodes of axillary metastasis of breast cancer. The micelles could release the drug in response to pH changes and inhibit tumor growth and axillary lymph node metastasis [67]. Studies have shown that small micelles (< 50 nm) can inhibit lymph node metastasis, reduce recurrence, and improve survival rates. A report demonstrated that even after systemic injection of platinum-based anticancer drugs in a homologous melanoma model, 50 nm polymer micelles can still target lymph node metastasis and inhibit the growth of metastatic lesions, whereas larger nanocarriers cannot penetrate the metastatic site [68]. Curcumin-loaded porphyrin micelles (T-MP) were successfully prepared with a size smaller than 50 nm using a surfactant-stripping method. These micelles were modified with trastuzumab antibodies and exhibited excellent photothermal conversion efficiency. They demonstrated targeted photothermal ablation of HT-29 colon cancer cells overexpressing HER2. Compared to non-targeted micelles (nonT-MP), T-MP exhibited enhanced accumulation in metastatic lesions of colonic mesenteric lymph nodes. Following surgical resection of primary tumors, minimally invasive photothermal therapy using T-MP significantly prolonged the survival of mice with metastatic lymph nodes. The therapeutic effect was comparable to or even better than that of traditional lymph node dissection, the experimental procedure is shown in Fig. 5A, where the temperature of the sentinel lymph node treated with T-MP increases from 27.3 °C to 54.3 °C, significantly higher than the non-T-MP-treated node (46.5 °C) (Fig. 5B). This observation can be attributed to the improved distribution of T-MP within the metastatic lymph node compared to non-T-MP. Furthermore, in metastatic lymph nodes subjected to T-MP-assisted photothermal therapy, a greater number of tumor cells exhibiting characteristics such as cellular shrinkage, nuclear fragmentation, and solidification, indicative of cell death, can be observed (Fig. 5C). In a lymph node metastasis mouse tumor model, tumor recurrence is significantly delayed and suppressed after T-MP treatment (Fig. 5D, E), leading to improved survival time (Fig. 5F) and changes in body weight (Fig. 5G) [69]. Hence, in the design of nanocarrier-based drug delivery systems, the size of the nanocarrier plays a critical role in its ability to reach metastatic sites.

### Drug delivery systems based on inorganic nanoparticles

Inorganic nanoparticles have been used as drug delivery carriers for various diagnostic and therapeutic applications due to their unique physical, electrical, magnetic, and optical properties. By relying on different sizes, structures, and precise ratios, inorganic nanocarriers can deliver therapeutic drug molecules to specific tumor sites through lymphatic vessels [70, 71]. With the assistance of special functions such as sound, electricity, and magnetism, nanodrugs can penetrate deep into tissues and exert unique anti-tumor effects through heat induction. Currently, commonly used inorganic materials include gold, iron, silica, and carbon, which will be discussed one by one below.

Gold nanoparticles have received widespread attention in the design of nanomedicine delivery systems for the lymphatic system due to their excellent drug-loading capacity, unique surface properties, and natural adjuvant effects [72]. Nanocarriers based on gold nanoparticles have diverse morphologies, such as spherical, rod-shaped, and nanocages, and their sizes can be adjusted as needed to facilitate better absorption by the lymphatic system. The role of gold nanoparticle carriers in cancer diagnosis and treatment mainly lies in their ability to effectively induce antigen-specific immune responses, thereby inhibiting tumor growth [73]. For example, amphiphilic gold nanoparticles coated with hexanethiol and 11-mercaptoundecanesulfonic acid was used for the delivery of TLR7 ligands, as an immune stimulant for tumor lymph nodes, which can cause local immune activation and stimulate cytotoxic T cells to respond to tumors [74]. Additionally, gold nanoparticle carriers can be combined with hyperthermia to inhibit tumor metastasis. For instance, neutral polyethylene glycol multialloy nanorods with a diameter of approximately 10 nm can be delivered to tumor cells in lymph nodes through lymphatic vessels, thereby achieving local photothermal therapy [75].

Iron oxide nanoparticles have been widely studied in nanomaterials research due to their inherent magnetism and paramagnetism, and have been applied in various fields such as electronics, environment, and biomedicine [76]. By precisely shaping the structural properties based on iron oxide nanoparticles, diagnostic and therapeutic drugs can be effectively delivered to the lesion site. Furthermore, the inherent magnetic and paramagnetic properties of iron oxide can be utilized to achieve specific drug delivery functions, thereby improving the therapeutic effect of drugs. The specific functions mainly include guiding drug delivery with the magnetism of the ions, solving the problem of drug control release in nanocarriers, and achieving synergistic magnetic hyperthermia therapy [77].

Ultra-small superparamagnetic iron oxide nanoparticles had faster lymphatic drainage rates, earlier accumulation in sentinel lymph nodes, and greater particle accumulation in lymph nodes when injected in the lymphatic system [78]. Iron oxide nanoparticles coated with lauric acid and human serum albumin (HSA) and adsorbed the anti-tumor drug mitoxantrone onto HSA, resulting in an average diameter of 7 nm. The resulting ions exhibited stronger stability and linear release kinetics within 72 h [79]. Furthermore, some research groups have prepared magnetic nanocarriers by coating them with temperature-sensitive polymers to achieve enhanced drug release through particle exchange in a magnetic field. For example, mesoporous magnetic ions were developed to load doxorubicin based on chitosan, which exhibited enhanced tumor therapy efficacy under an alternating magnetic field [80]. Lipid-polyethylene glycol-coated iron oxide nanoparticles that continuously released doxorubicin and could raise the temperature to 43^◦^C under magnetic field intervention to produce sufficient heat, achieving effective combination of chemotherapy and hyperthermia [81]. This is an effective approach that utilizes the magnetic and biological properties of ions to bind or load drugs onto nanoscale iron oxide carriers, thus improving the therapeutic efficacy of drugs.

Mesoporous silica nanoparticles (MSNs) have attracted significant attention in cancer vaccine, adjuvant design, and cancer therapy due to their controllable pore structure, surface modifiability, and biocompatibility. MSNs with mesoporous structures have high drug loading capacity, which can enhance the immunogenicity of antigens. For example, mesoporous silica rods (MSRs) were designed to adsorb polyethyleneimine (PEI) to enhance the immunogenicity of antigens. After one injection of the MSR-PEI vaccine containing E7 peptide, 80% of large tumor lesions were cleared in the TC-1 mouse tumor model, and long-lasting memory immunity was induced [82]. MSNs can also be used for combined immunostimulation and drug delivery to enhance anti-tumor therapy. Further, a biodegradable dendritic mesoporous organosilica nanoparticle was reported that can transport antigen protein OVA and TLR9 agonist to APC and induce endosomal escape [83].

Carbon-based nanomaterials have great potential in lymphatic drug delivery due to their large internal space for high drug loading and the ability to provide personalized active functional groups through chemical covalent modification [84]. Currently, graphene nanoparticles and carbon nanotubes are widely used as carbon-based nanodelivery materials. For instance, polyethylene glycol-modified graphene nanoparticles serve as a highly modular and biodegradable antigen vaccine platform that can quickly and efficiently accumulate (15–20%ID/g) in lymph nodes and persist for a long time. According to the report, carbon nanotubes and loaded gemcitabine into magnetic multi-walled carbon nanotubes with diameters of 40–60 nm, producing nanotubes with dual functions of carbon-based materials and Fe_3_O_4_. These nanotubes can reduce cancer lymphatic metastasis and achieve stronger anticancer therapy under magnetic guidance [85].

### Nanoparticle drug delivery systems based on hydrogels

Hydrogels are three-dimensional networks composed of crosslinked hydrophilic polymers, and are widely used in drug delivery and tissue engineering due to their biodegradability, drug release effects, and excellent biocompatibility [86]. In particular, newly developed injectable hydrogels, which can be administered in situ, have been widely studied due to their advantages such as reducing systemic adverse reactions, drug dosage, and targeting of lesions [87]. Previous studies have found that drug nanocarriers with excellent lymphatic system targeting properties can be prepared based on hydrogels with reasonable materials and ratios [88]. A cholesterol-poly(ethylene glycol)-poly-l-lysine nanogel based on a synthetic long peptide antigen could effectively present CD8^+^ T cell antigens and inhibit tumor growth after subcutaneous injection in mice [89].

The application of hydrogel nanoparticles as drug delivery carriers in the lymphatic system mainly involves inducing immune therapy of humoral and cellular responses and antigen presentation, recruiting immune cells to guide the design of anti-tumor vaccines. For example, the covalently connected small molecule TLR7/8 agonists of imidazoquinoline to degradable polymer hydrogel nanoparticles with a diameter of 50 nm. The prepared nanogel combined the effective triggering of TLR7/8 with local injection sites and immune activation of draining lymph nodes, maintaining the unchanged activation performance of TLR7/8 in vitro. After intratumoral injection, the immune-stimulating nanogel could enhance the therapeutic effect of locally applied imidazole drugs [90]. The targeting of hydrogel nanoparticles could be significantly improved through polyethylene glycol and proposed a design strategy for polymer hydrogel nanoparticles that could effectively target multiple immune cell subpopulations within lymph nodes. The initiation of antigen-specific T cells in lymph nodes increased with the accumulation of nanoparticles in lymph nodes. Based on the excellent lymphatic drainage effect of polyethylene glycol, polyethylene glycol-polymethyl acrylic acid nanoparticles can efficiently deliver peptides, improving antigen presentation capability [91].

### Novel nanocapsules

Nanocapsules are a newly developed lymphatic system nanodrug delivery carrier in recent years. Due to the controllable capsule size, distribution particle size, and good biocompatibility, they have been widely used in the development of lymphatic targeted drug delivery platforms for drug control release and antigen and adjuvant delivery [92, 93]. Research found that small-sized (100 nm) polyamino acid nanocapsules have better lymphatic absorption and biological distribution characteristics. Using 100 nm nanocapsules, a highly loaded and sustained-release paclitaxel nanocarrier can be prepared. At the same time, nanocapsules can form a storage pool at the injection site, with slow lymphatic drainage and long lymphatic retention time [94]. In addition, lipid nanocapsules can effectively promote APC lymphatic drainage transport and significantly improve the therapeutic effect of tumor vaccines by loading protein or peptide antigens [95].

### Biomimetic delivery system

With the deepening of research, many biomimetic nanocarriers have emerged in targeted lymphatic system nanocarriers, mainly including cell membrane-derived nanovesicles, DNA nanocage based carriers, which not only have good targeting properties but also have broad prospects in immune activation [96]. DNA nanoparticles targeted to Langerhans cells have been shown to have good immune cell recruitment and induction of cellular immunity. For example, a new structure–function relationship was used to prepare a pH-sensitive cationic lipid nanoparticle with a suitable combination of hydrophilic head groups and hydrophobic tails, which could promote liver cell targeting and endosome escape and affect the use of siRNA [97].

**Fig. 6:**
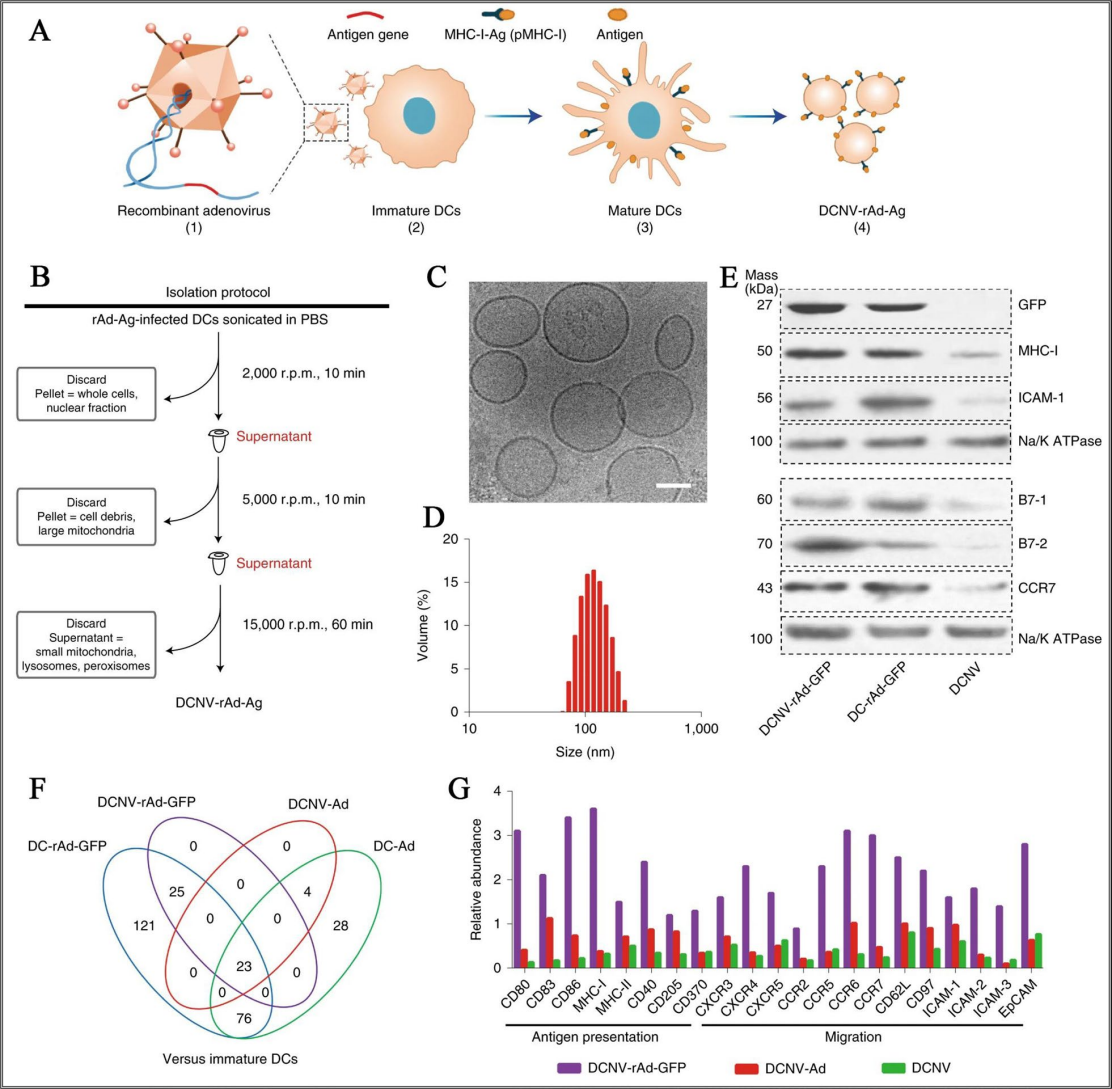
Design and partial characterization of gene-engineered cell membrane nanovesicles for integrated antigen self-presentation and immune checkpoint blockade. **A** Generation of DCNVs derived from adenovirus-infected mature dendritic cells. **B** Schematic illustration of the generation of DCNV-rAd-Ag. **C** Cryo-electron microscopy. **D** Dynamic light scattering analyses. **E** The western blot on membrane proteins from DCNV-rAd-GFP. **F** Comparison of upregulated immune-response-related proteins in NVs and DCs. **G** The relative abundance of antigen presentation and migration-related proteins on DCNV-rAd-GFP [100]. Copyright © 2022 The Authors

However, due to the insufficient number of tumor-specific effector T cells in the patient's body and the inhibitory effect of the tumor immune suppressive microenvironment on immune cells, not all patients respond to immunotherapy. To address this issue, cell membrane-derived nanovesicles were developed to as a new kind of lymph node targeting nanocarriers. As reported, *E. coli* membrane was utilized to prepare as nanocarrier to wrap gold nanoparticles with a particle size of about 40 nm to prepare an antibacterial vaccine. When delivered by subcutaneous injection to the drained lymph nodes of mice, it can induce rapid activation and maturation of DCs, resulting in a robust antibody response and Th1- and Th17-based cellular responses to *E. coli* [98]. This combined application strategy provides a possibility for drug encapsulation and vaccine delivery using cell membrane collected from immune cells such as macrophages, B cells, dendritic cells, and NK cells, and provides a promising strategy for inhibiting cancer development and metastasis by inducing or modulating immune responses [99]. As shown in Fig. 6. engineered cell membrane nanovesicles were constructed for integrated antigen self-presentation and immune-inhibitory reversal. In this study, the authors generated recombinant adenoviruses expressing membrane-targeted modified green fluorescent protein (rAd-GFP) or ovalbumin (rAd-OVA) by infecting dendritic cells (DCs) with the viruses. Immature DC2.4 cells were transduced with the viruses to express membrane-targeted model antigen GFP and differentiate into mature DCs (Fig. 6A). Finally, DCNV-rAd-Ag was isolated using multiple-step density gradient ultracentrifugation, resulting in the production of 37.43 ± 6.68 mg of dendritic cell nanovesicles (DCNVs) (Fig. 6B). These DCNVs exhibited uniform vesicular morphology (Fig. 6C) with an average diameter of approximately 108 nm (Fig. 6D). The obtained DCNV-rAd-Ag showed similar levels of major functional membrane proteins on its surface compared to the parental DC-Rad-Ag cells (Fig. 6E). Mass spectrometry analysis revealed significant upregulation of proteins on DCNV-rAd-GFP, as well as upregulation of various co-stimulatory molecules such as CD80, CD86, and CD40, which play important roles in antigen presentation and enhancing immune responses (Fig. 6F, G) [100].

**Fig. 7:**
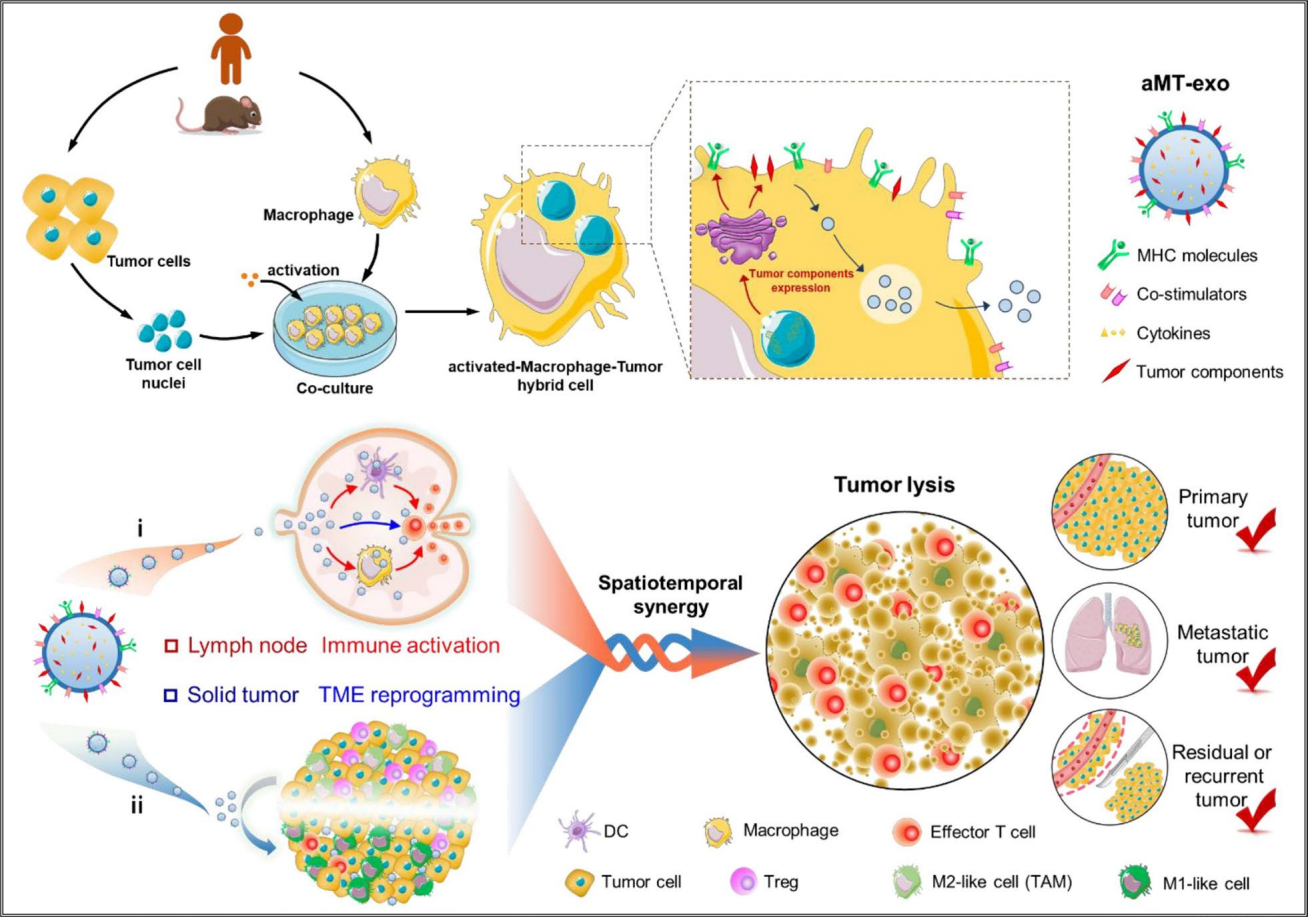
Preparation and anticipated tumor inhibition mechanism of macrophage-tumor chimeric exosomes. Biologically reprogrammed macrophage-tumor chimeric exosomes were constructed by introducing tumor nuclei into activated macrophages (aMT-exos). This strategy generated exosomes with tumor components as well as classical activated macrophage (M1) properties, including tumor-antigen-MHC molecules, co-stimulatory molecules, immune-activated cytokines and other tumor components [101]. Copyright © 2021 American Association for the Advancement of Science

Furthermore, a hybrid exosomes (aMT-exos) were also designed by incorporating isolated tumor cell nuclei into activated M1-like macrophages. Experimental evidence demonstrated that aMT-exos could accumulate in lymph nodes and various tumors in xenograft mice. Moreover, they were capable of inducing T cell activation in lymph nodes through both the classical antigen presentation by cells and a unique "direct exosome interaction" mechanism (Fig. 7) [101]. Indeed, the application of these novel nanoscale lymphatic delivery systems primarily focuses on new therapies for acquired immunodeficiency syndrome (AIDS) and antiviral treatments. In the context of tumor diagnosis and treatment, research and design studies are relatively limited and mostly at the stage of basic animal research. However, it is possible that this area will become a major research hotspot in the future. As scientists continue to explore and understand the potential of these systems, there may be advancements in utilizing them for tumor-related applications. In summary, we summarized a variety of typical nanosystems for lymph node delivery diagnosis or treat disease in recent years (Table 1).

**Table 1:** Diagnosis and treatment nanoparticle delivery system for lymphatic system

Nanoplatform	Nanoparticle size	Encapsulation/Payload/Components	Target sites	Lymphatic targeting system	A.R	Disease models	Application	Ref
Liposomes	129 ± 5 nm	siRNA	CD4^+^ T cells	Internalisation	i.v	/	Systemic Gene Silencing; Targeted therapies	[53]
Liposomes	20–30 nm	Neoantigens; Vaccine adjuvant	Lymph nodes	Cell-mediated trafocation via DCs	s.c	Melanoma (B16-F10)	Vaccination; Antitumor immunotherapy	[58]
Micelle polymers	20 ± 5 nm	DOX	Lymph nodes	Passive targeting	s.c	Melanoma (A375)	Lymph node metastasis and antitumor chemotherapy	[63]
Poly(lactide-co-glycolide) microparticles	300 ± 21 nm	PolyIC	APCs in Lymph nodes	Cell-mediated trafocation via APCs	i.LN	/	Vaccination; Antitumor immunotherapy	[66]
Micelle polymers	55 nm	Epirubicin	Metastatic axillary lymph nodes	Passive targeting	i.v	Breast cancer (MDA-MB-231-D3H2LN)	Suppression of axillary lymph node metastasis	[67]
Micelle polymers	30 nm	Platinum	Lymph nodes	Passive targeting	i.v	Melanoma (B16-F10)	Non-invasive management of nodal disease	[68]
Micelle polymers	40 nm	Curcumin Trastuzumab	Metastatic mesenteric sentinel lymph nodes	Active targeting via igands	i.v	Colorectal cancer (HT-29)	Lymph Node Metastasis Homing and Photothermal Therapy in Orthotopic Colorectal Tumor Model	[69]
Gold NPs	5 nm	TLR7 ligand	Lymph nodes	Passive targeting	s.c	Colon carcinoma	Antitumor immunotherapy	[73]
Polymeric gold nanorods	1066 nm	Polyethylene glycol	Lymph nodes	Passive targeting	i.LN	MXH10/Mo/lpr mice	In vivo NIR fluorescence imaging; Anti-tumor effect of PTT	[74]
Outer membrane vesicles	41.9 ± 0.9 nm	Gold NPs	DC	Cell-mediated trafocation via APCs	s.c	/	Anti-bacterial vaccination	[75]
Iron oxide NPs	15–58 nm	/	Lymph nodes	Endocytosis and pinocytosis	s.c	/	In vivo optical imaging	[77]
CNTs and carbon NPs	40–60 nm 8–12 nm	/	Lymph nodes	Internalisation	s.c	Pancreatic cancer (BxPC-3)	Magnetic lymphatic targeting drug-delivery system; Treatment of tumour metastasis	[84]
Hydrogels	50 nm	Imidazoquinoline	Draining lymph nodes	Passive targeting	s.c	/	Antitumor immunotherapy	[89]
Hydrogels	200 nm	Polyethylene glycol	DC	Lipid raft-mediated trafocation via DCs	s.c	OT-1 mice	The lymphatic delivery of antigens	[90]
Nanocapsules	242 ± 6 nm	Polyglucosamine/Squalene	Popliteal lymph node	Cell-mediated trafocation via APCs	s.c	/	Immunostimulating	[93]
Lipid nanocapsules	/	Ovalbumin/Polyinosinic-polycytidylic acid/ Monophosphoryl lipid A	Draining lymph nodes	Cell-mediated trafocation via APCs	Intratracheal administration s.c	OT-1 mice	Pulmonary Nanoparticle Vaccination	[94]
Chimeric exosomes	30–150 nm	M1-like Macrophage; E.G7 Tumor cell nuclei	Lymph nodes; Tumors	Passive targeting	Intratumoral injection	Lymphoma;Breast cancers; Melanoma	Activation in the immune response and the tumor microenvironment	[101]

## Nanoparticle for lymph node specific imaging and identification

For most malignant tumors, the presence of lymph node metastasis is of great significance in guiding treatment and improving prognosis [102]. Developing a reliable lymph node identification technique to evaluate lymph node metastasis in cancer patients and guide complete lymph node dissection is a research hotspot to improve long-term surgical treatment outcomes. To accurately image, identify and evaluate lymph node function, researchers have developed various lymphatic nanocarriers for imaging, such as liposomes, dendrimers, quantum dots, and nanobubbles, based on nanotechnology. This section will elaborate on the design and application of these nanocarrier materials.

### Liposomes

Liposomal nanocarriers have been used not only for cancer diagnosis and immune-activating therapy but also as delivery agents for imaging agents in lymphatic system applications. Many imaging agents can be covalently or non-covalently bound to different compartments of liposomal nanocarriers and hydrophilic dyes can also be encapsulated within the aqueous interior. At the same time, receptor or molecule attachment to the surface of liposomal nanocarriers can be used to construct molecular probes with targeting properties [103, 104].

For lymph node imaging using liposomal nanocarriers, commonly used imaging agents are ^99m^Tc or blue dyes. For example, Osborne prepared liposomes labeled with ^99m^Tc for lymphatic imaging [105]. However, subsequent studies have shown that ^99m^Tc-labeled liposomal nanocarriers have poor stability and are not conducive to long-term stable labeling of lymph nodes in vivo [37]. To solve the problem of poor stability, blue dye liposomes were later invented for long-term imaging of lymph nodes. A blue dye-encapsulated liposomes as a potential system could maintain blue staining in lymph nodes for more than a week after subcutaneous injection in living animals [106]. The team further prepared blue liposomes containing glutathione by combining ^99m^Tc with blue dye, which could be traced in both directions by lymphatic channels and radiation detection instruments, and used for lymphatic scintigraphy and intraoperative lymph node identification [107]. However, clinical trials of this system are essential to further demonstrate their efficacy.

### Dendrimers

Dendrimers are highly defined, branched macromolecules with high-end functional groups and compact molecular structure, produced through cascade branching. The asymmetrical polyamide nanofilm has two layers: a porous layer of spherical polyamide dendrimers at the bottom and a dense layer of polyamide at the top, with highly ordered nanopore structure [108]. After several generations of development, geometrically enclosed nanoscale structures with host–guest container characteristics have been formed. These nanostructures have different internal composition components such as carbon, nitrogen, silicon, sulfur, phosphorus, or metals, leading to their widespread attention in biomedical functions, especially for cancer diagnosis and imaging [109].

**Fig. 8:**
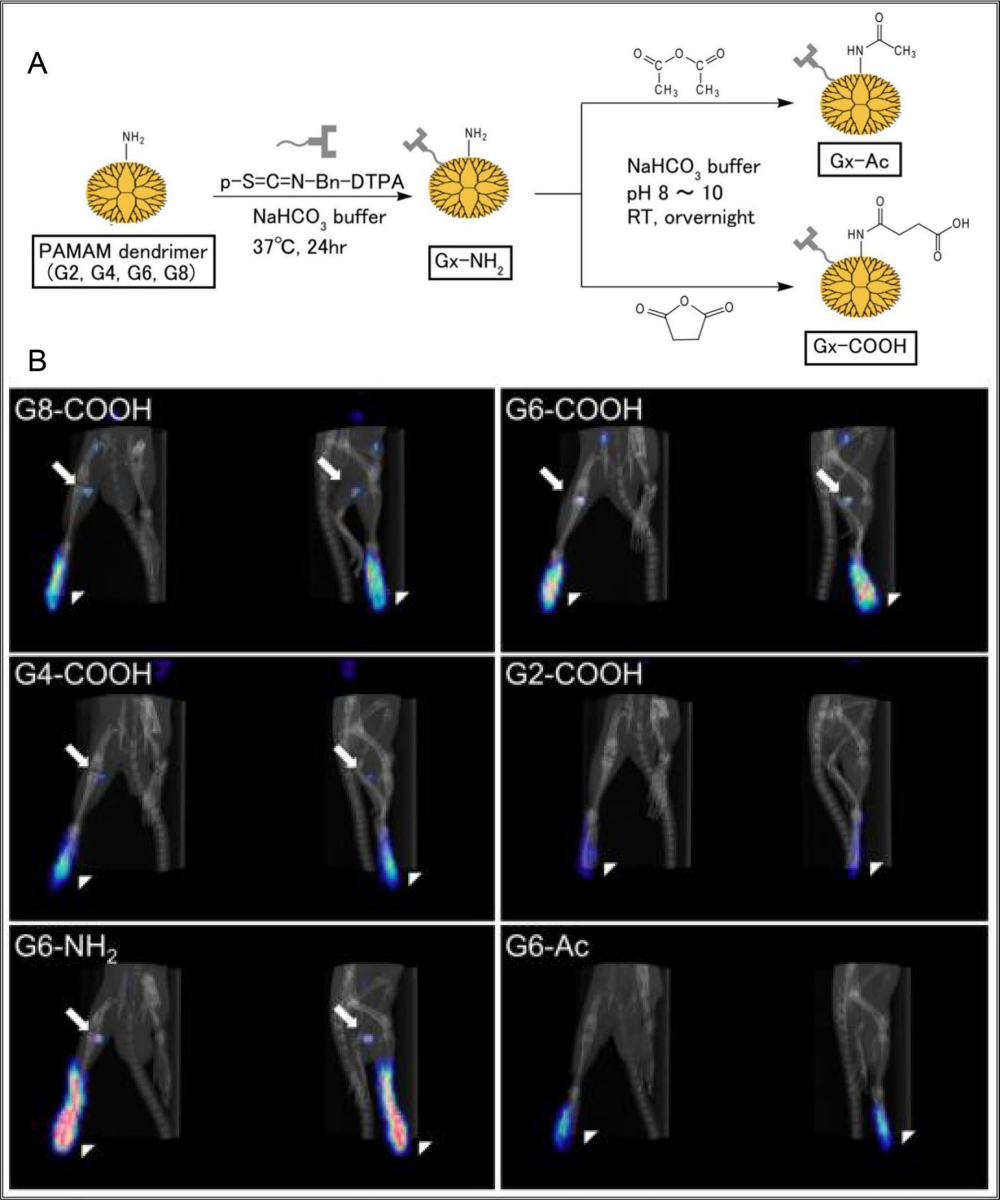
Synthesis scheme of the 12 types of dendrimer for biodistribution assay and SPECT imaging (**A**), and the fused SPECT/CT images of radiolabeled dendrimer-injected rats after 24 h. Left and right panels are anterior and lateral views, respectively. Arrows and arrow heads indicate the SLN and the injection site, respectively (**B**) [112]. Copyright © 2015 Elsevier Inc

In cancer lymph node imaging and identification, dendrimers are mainly used for magnetic resonance imaging, fluorescence imaging and SPECT/CT imaging. For example, Talanov prepared a dual-purpose dendrimer for MRI and fluorescence imaging using PAMAM dendrimers covalently linked with GdIII-DTPA chelates and near-infrared fluorescent dye Cy5.5. The dendrimer nanocarrier can provide clear MRI and fluorescence images of lymph nodes and locate lymph nodes and their related lymphatic channels. This study effectively demonstrates the huge potential of dendrimers as a platform for developing magnetic resonance and optical imaging of the lymphatic system [110]. Waldmann also demonstrated that PAMAM-G6 dendrimers are one of the best MRI imaging agents, even in the imaging identification of metastatic lymph nodes [111]. Niki [112] determined the optimal dendritic polymer structure for sentinel lymph node (SLN) imaging through the preparation of 12 different generations (G2, G4, G6, and G8) and different terminal groups (amino, carboxyl, and acetyl) (Fig. 8A). In his study involving intradermal injection of radiolabeled dendritic polymers into the right foot of rats, it was found that all G2 dendritic polymers primarily accumulated in the kidneys. Dendritic polymers with amino, acetyl, and carboxyl end groups larger than G4 were mainly distributed in the injection site, blood, and SLNs. Within the SLNs, macrophages and T-cells were unable to recognize dendritic polymers with carboxyl end groups. Finally, successful imaging detection of SLNs was achieved through single-photon emission computed tomography using dendritic polymers with carboxyl end groups larger than G4 (Fig. 8B). Subsequent in-depth studies have found that the unique diagnostic characteristics provided by dendrimers have played a unique role in the development of many biomedical applications. Their applications in proteomics clearly demonstrate their enormous potential in developing new biomedical devices and methods for human disease imaging and identification, especially in potential applications for lymph node imaging.

### Quantum dots

Quantum dots are nearly spherical semiconductor particles with a diameter of approximately 2–8 nm, typically composed of atoms from the II-VI or III-V groups of the periodic table. Due to their unique semiconductor properties and size-dependent fluorescent properties, quantum dots have become an attractive diagnostic and imaging material. Compared to current diagnostic techniques, nanocrystals based on quantum dots can provide a long-lasting, strong, and stable fluorescent signal at a low cost, and also have strong resistance to photobleaching [113]. Quantum dots can be covalently linked to antibodies, peptides, nucleic acids, or other ligands, endowing them with unique targeting capabilities [114].

As a semiconductor material, one of the earliest in vivo research applications of quantum dots is mainly for the localization of the reticuloendothelial system (RES) and lymph nodes, mainly used for near-infrared fluorescence imaging. A kind of near-infrared quantum dots with an emission wavelength of 850 nm was used for sentinel lymph node localization. In this study, real-time tracking was performed by injecting quantum dots subcutaneously into live mice, and it was found that sentinel lymph nodes could be located 1 cm below the skin surface, reducing the surgical incision guided by conventional radioactive labeling for sentinel lymph node removal [115]. In recent years, more and more research has been conducted to explore different materials that can be used to prepare the surface or core of quantum dots. For example, quantum dots with a core/shell/shell structure composed of InAsxP_(1-x) alloy, InP, and ZnSe, showed tunable emission in the near-infrared region and have been successfully used in sentinel lymph node localization experiments in various animal models [116]. However, all of these studies are based on quantum dots as a technological hypothesis and animal model verification, and further validation is needed for their lymph node imaging detection effects.

### Other nanomedicines use to lymph node imaging

**Fig. 9:**
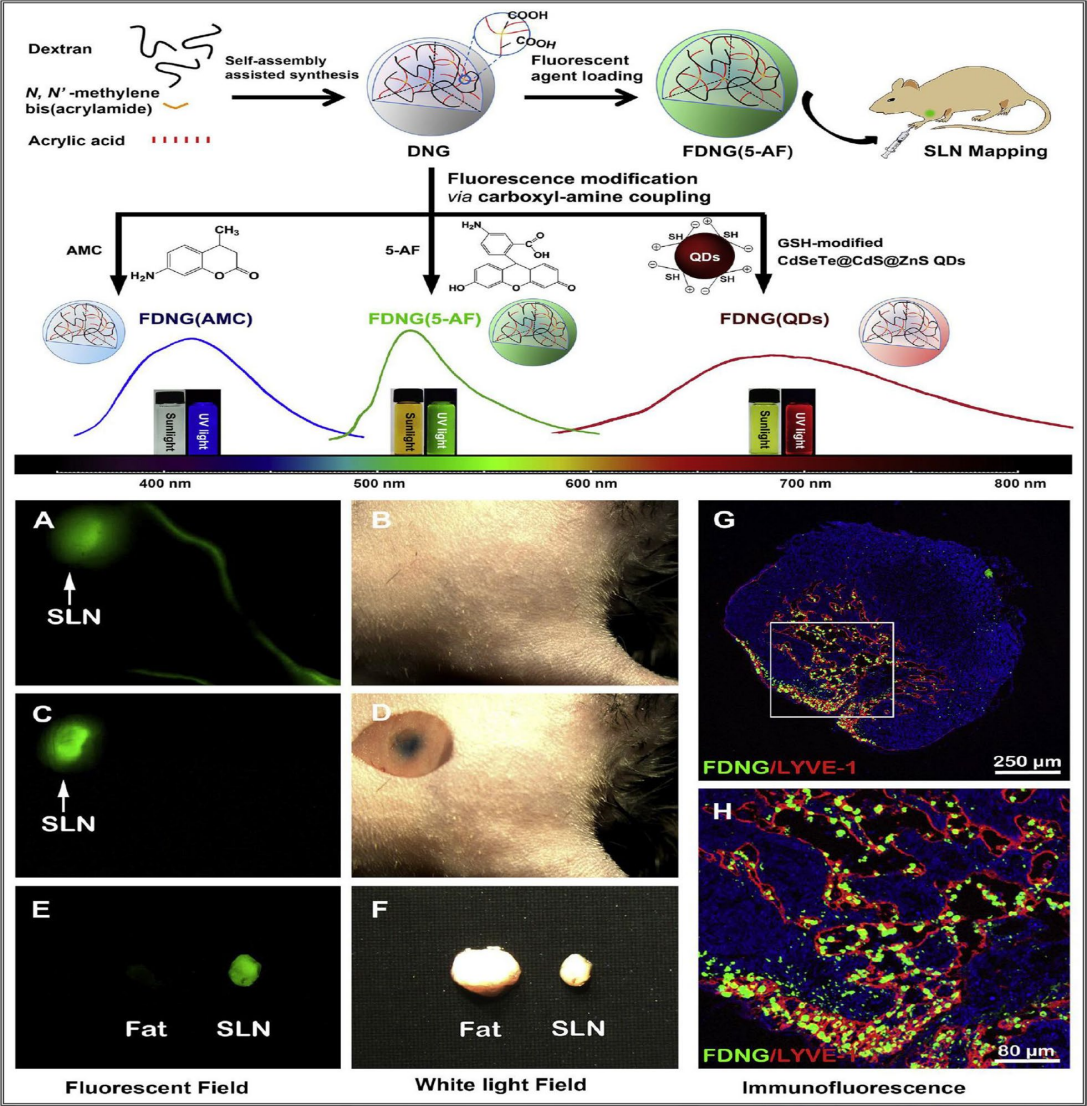
Simplified preparation of nanogel, modified spectral analysis of fluorescent agent, and imaging of mouse axillary lymph nodes before and after front limb injection. Fluorescent (**A**) and optical (**B**) images of FDNG (5-AF) selectively entering LVs and SLN. Fluorescent (**C**) and optical (**D**) images of FDNG(5-AF) and methylene blue co-injected mice after skin removal. Fluorescent (**E**) and optical (**F**) images of dissected SLN and adjacent fat tissues. **G** Immunohistofluorescence staining of dissected SLN from FDNG(5-AF) treated mouse after 12 h of injection. (**H**) The partially enlarged image of (**G**) [117]. Copyright © 2014 Elsevier Ltd

Due to the unique functions of nanoscale imaging probes, they can be adapted to different imaging modes, making them an ideal system for enhancing lymph node identification and localization. Currently, many types of nanoscale particles for lymphatic system imaging have been reported, such as fluorescent nanogels (Fig. 9), which serve as highly efficient molecular imaging probes that can specifically, sensitively, stably, non-invasively, and safely locate sentinel lymph nodes, thus predicting tumor metastasis and guiding therapy. In the mouse model study, the results showed that FDNG rapidly and selectively diffused to the left lung and left anterior descending branch less than 1 min after injection (Fig. 9A, B). When methylene blue was injected into the same site, the green fluorescence overlapped with the blue dye, allowing for accurate localization of the SLNposition (Fig. 9C, D). In ex vivo imaging, a strong fluorescent outline of the SLN was visible, while adjacent adipose tissue did not exhibit fluorescence (Fig. 9E, F), indicating high specificity of FDNG for SLN diagnosis and ensuring the accuracy of SLN biopsy. Additionally, further immunofluorescence staining demonstrated the accumulation of abundant FDNG within the SLN in the left lung, providing direct evidence of FDNG's specific uptake by SLN (Fig. 9G, H) [117]. In addition to the above-mentioned nanodelivery materials, other major ones include radiopharmaceutical colloid nanoparticles and ultrasound nanobubbles.

Radiopharmaceutical colloids were first proposed in 1955 and were primarily used for preoperative treatment of breast cancer patients [118]. As research progressed, radioactive gold was gradually replaced by ^99m^Tc due to its easy availability, low cost, and excellent imaging properties [119]. The size range of standard radioactive nanocolloids is 10 nm to 1000 nm, and they have high potential for clinical translation. Reported lymph node targeting agents include sulfur colloid labeled with ^99m^Tc, as well as other agents such as ^99m^Tc-labeled dextran, ^99m^Tc-hydroxyethyl starch, and ^99m^Tc-human serum albumin [120]. However, none of these fully meet the criteria for an ideal imaging agent, and the search for the best nanocolloid radiopharmaceutical continues.

Ultrasound is the most common biomedical imaging modality, and ultrasound contrast agents are mainly used to increase the reflectivity or backscatter of blood and tissue. Nano-bubbles, as a new type of nanocarrier, are currently being widely studied in combination with ultrasound for identification of sentinel lymph nodes [121]. The preparation material of nano-bubbles mainly consists of a double-layered shell of perfluorocarbon nanodroplets or albumin and an inner layer of a biodegradable polymer called polyethylene glycol. This shell-core structure can effectively encapsulate gases such as nitrogen for tracking using ultrasound imaging techniques [122, 123]. During the ultrasound imaging process, since nano-bubbles can rupture and form smaller bubbles, some have suggested using nano-bubbles to carry therapeutic drugs and injecting them into cells through the explosion of the nano-bubbles to achieve drug delivery and controlled release in lymph nodes. However, this process can cause thermal tissue damage, and its feasibility needs further study [124]. We also summarized a variety of typical nanomedicine for lymph node imaging and identification in recent years (Table 2).

**Table 2:** Nanoparticle of lymph node imaging and identification

Nanoplatform	Nanoparticle size	Imaging methodology	Imaging sites	Imaging agents	A.R	Encapsulation/Coating/Component	Features	Ref.
Liposomes	33 nm	γ-camera imaging	Regional lymph nodes	^99m^Tc	s.c	/	Lymphatic scintigraphy	[103]
Liposomes	136 nm	γ-camera imaging	Popliteal nodes	^99m^Tc	s.c	Biotin; Avidin	The delivery of chemotherapeutic drugs, vaccine antigens, and biologic agents to lymph nodes	[104]
Liposomes	115.3–148.9 nm	γ-camera imaging	Sentinel lymph nodes	^99m^Tc Blue dye	s.c	Blue dye; Glutathione; Biotin	Lymphatic scintigraphy and intraoperative lymph node identification	[105]
PAMAM Dendrimers	/	MRI-FI	Sentinel lymph nodes	GdIII-DTPA Cy5.5	s.c	/	Magnetic resonance and optical imaging of the lymphatic system	[108]
PAMAM -G6 Dendrimers	9 nm	MR	Sentinel lymph nodes	GdIII	Peritumoral injection	/	Localization of sentinel lymph nodes in human breast cancer	[109]
Quantum Dots	10 nm	FI	Sentinel lymph nodes	NIR QDs	s.c	Oligomeric phosphine	Direct visual guidance throughout the entire SLN mapping procedure	[113]
Quantum Dots	15 nm	FI	Sentinel lymph nodes	NIR QDs	s.c	InAsxP1-x/InP/ZnSe	NIR Sentinel lymph node mapping	[114]
Microbubbles	50–100 nm	Contrast-enhanced ultrasound	Sentinel lymph nodes	Microbubbles	i.d	Sulfur hexafluoride	Sentinel lymph node identification in early-stage breast cancer	[119]
Nanocarbon	/	Endoscopy	Sentinel lymph nodes	Nanocarbon suspension	Intrathyroid injection	/	Clear lymph node clearance in PTC patients; Decrease in parathyroid damage	[123]
Nanocarbon	150 nm	Endoscopy	Lymph nodes	Nanocarbon suspension	Submucosal injection	/	Tracking lymph node metastases of colorectal cancer	[124]
Nanoparticles	/	PET/CT MRI	Lymph nodes	^68^ Ga-PSMA Ferumoxtran-10	i.v	PSMA Ferumoxtran-10	Diagnosis of Lymph Node Metastases in Prostate Cancer Patients	[125]
Nanotracer agents	7 nm	γ-camera imaging	Sentinel lymph nodes	^99m^Tc	i.d	Mannose DTPA	Melanoma Sentinel Lymph Node Mapping	[128]
Ultrasmall silica NPs	10 nm	PET-FI	Sentinel lymph nodes	^124^I Cy5/Cy5.5	s.c	PEG; CRGDY NIR dyes	Detection of tumor-carrying lymph nodes in the intraoperative setting	[129]

Although current studies report that a variety of nanocarriers, including liposome-based nanoparticles, micelles-based nanoparticles, inorganic nanoparticles, hydrogel nanoparticles, and orther novel nanoparticles, can all achieve lymph node targeting, and each carrier has its unique advantages. However, liposome-based nanoparticles are seem like the most promising for lymph node targeting delivery due to several unique characteristics. Firstly, liposome nanoparticles have good biocompatibility and high safety, making them excellent drug delivery carriers. Therefore, drugs based on liposomes are easy to be clinically translated. Secondly, liposomes have strong modifiability. The surface of liposomes can be modified with ligands or antibodies to enhance targeting to specific cell types or tissues. In addition, their size, charge, and other properties can be adjusted to improve their ability to accumulate in the lymphatic system. Moreover, liposome nanoparticles can also accumulate in the lymph nodes through passive targeting effects. For example, one study showed that LNPs based on lipid molecule 113-O12B could specifically target lymph nodes, which was significantly stronger than the targeting ability of LNPs based on lipid molecule ALC-0315 produced by Pfizer/Biotech [58].

While liposome-based nanoparticles show great promise for lymph node targeting delivery, other types of nanoparticles also offer potential benefits and could be chosen depending on the specific circumstances. Further research is needed to determine the most effective nanocarrier for this purpose.

## Clinical application status of nanomedicine for lymphatic system delivery

Despite substantial progress in lymphatic drug delivery in recent years, there are relatively few nanomedicines on the market that achieve pharmacokinetics or therapeutic effects through lymphatic delivery. So far, the most extensively studied polymer is polyethylene glycol ester, several of which have been approved by regulatory agencies. For example, in 1994, pegylated asparaginase (Oncaspar) for the treatment of acute lymphoblastic leukemia was approved by the FDA. However, most macromolecular bioproducts and drug delivery systems are developed for the treatment of cancer or inflammatory diseases, and clinical drugs for the lymphatic system are relatively scarce.

**Fig. 10:**
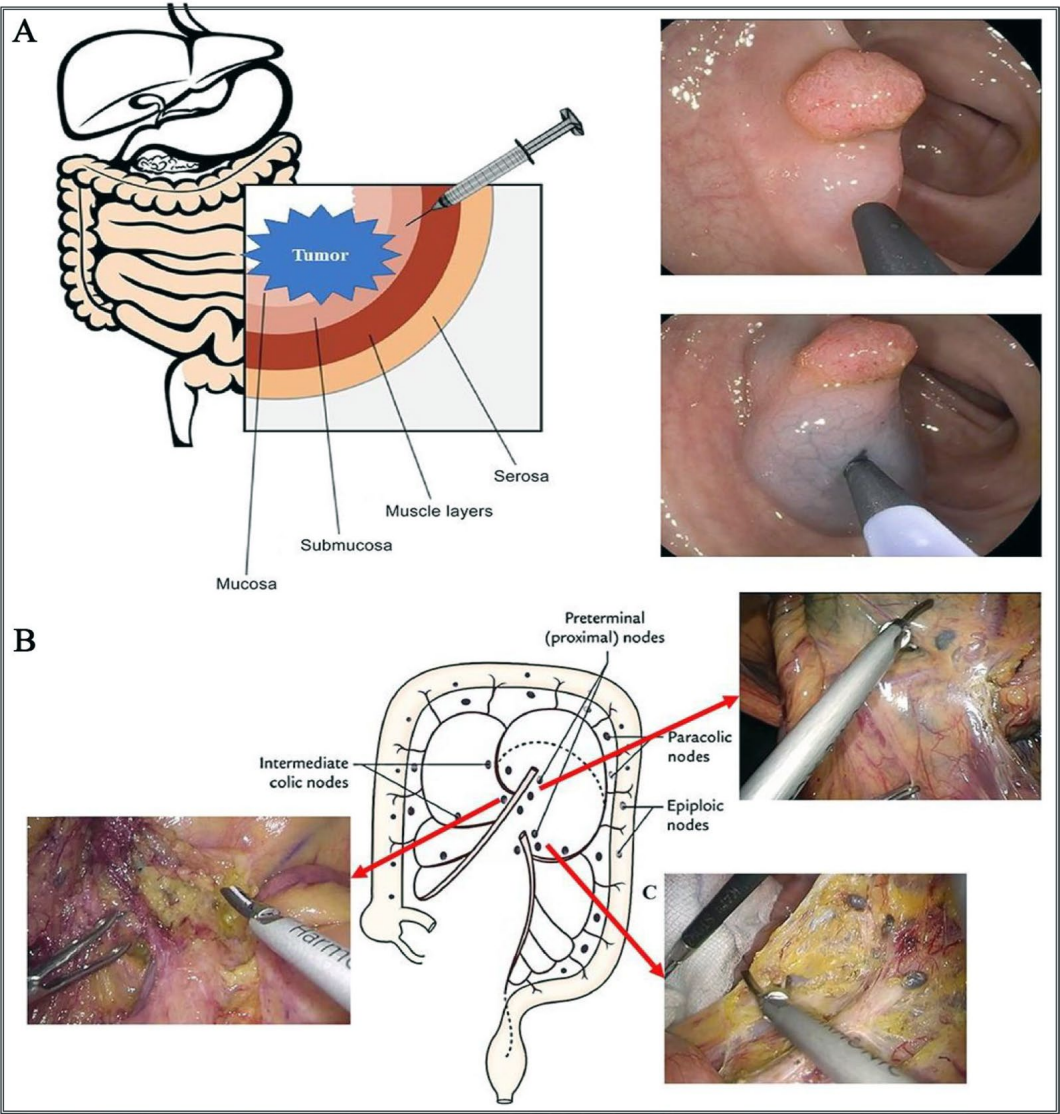
Schematic illustration of intramesorectal injection of nanocarbon (**A**) and intraoperative imaging and clearance images of mesorectal lymph nodes during surgery (**B**) and anterior resection of rectum (**C**) [126]. Copyright © 2020 Foundation of Clinical Oncology

Lymph node biopsy and dissection is a routine method for various cancer surgeries, such as thyroid cancer, breast cancer, colorectal cancer, and lung cancer. Currently, there are few nanomedicines used for biopsy and intraoperative lymph node dissection, mainly nanocarbon. For example, nanocarbon was utilized in endoscopic examination of thyroid cancer patients to clinically trace VI grade sentinel lymph nodes and protect the parathyroid glands. The results showed that nanocarbon, as a lymph node tracer, could better evaluate and allow for clearer lymph node clearance in papillary thyroid carcinoma patients [125]. Another research found that through a randomized controlled study (Fig. 10) that carbon nanospheres suspension is safe as a colon and rectal cancer tracer. More importantly, nanocarbon can significantly increase the number of detected lymph nodes in colorectal cancer, helping to improve the accuracy of lymph node staging and even improve patient survival [126].

**Fig. 11:**
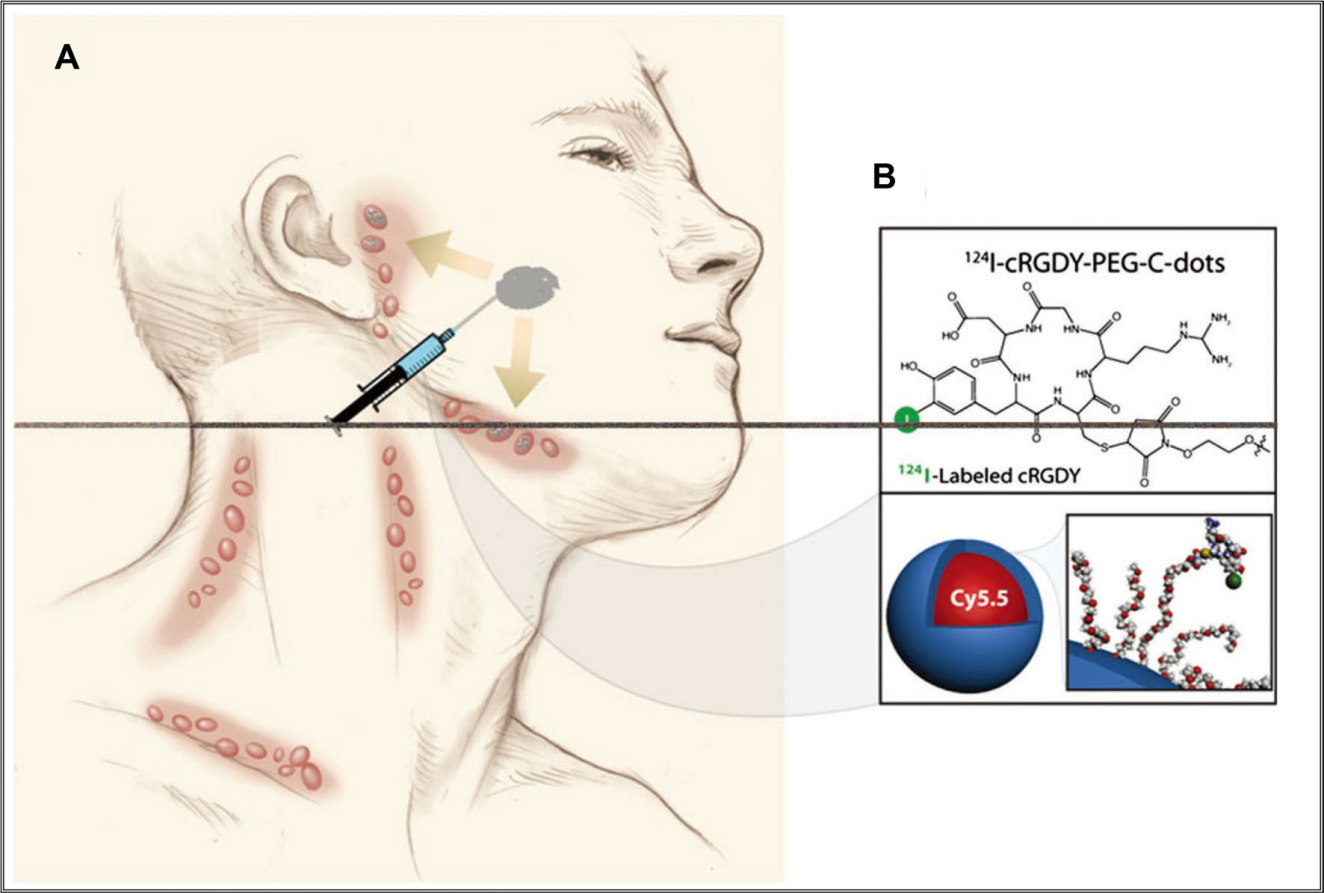
Schematic of SLN mapping in the head and neck using ^124^I-cRGDY-PEG-Cdots. **A** Injection of ^124^I-cRGDY-PEG-C dots about an oral cavity lesion with drainage to preauricular and submandibular nodes. **B** ^124^I-cRGDY-PEG-ylated core–shell silica nanoparticle with surfacebearing radiolabels and peptides and core-containing reactive dye molecules (insets) [129]. Copyright © 2013 The Royal Society of Chemistry

Other nanomedicines used for lymph node imaging include avidin-methotrexate anti-10-nanoparticles. A research found through a study of 45 patients with primary or recurrent prostate cancer that prostate-specific membrane antigen PET/CT and avidin-methotrexate anti-10-nanoparticle-enhanced MRI (nano-MRI) can both identify suspicious sentinel lymph nodes missed by another method. However, nano-MRI seems to be superior in detecting smaller suspicious LNs [127]. Furthermore, there are radioactive nanotracer agents such as ^99m^Tc sulfur colloid or the newer molecular imaging tracer ^99m^Tc-tilmanocept, which are used for lymph node imaging. Additionally, tumor-targeted ultra-small hybrid C dots have been utilized for this purpose. Relying on these types of nanotracer agents enables the acquisition of structural and functional information about lymphatic drainage [128]. For example, a silica-based nanohybrid platform composed of fluorescently labeled ^124^I cRGDY-PEG-C dots [129], consisting of near-infrared dye molecules, PEG, and cyclic arginine-glycine-aspartate peptide (CRGDY), has received FDA Investigational New Drug (IND) approval. Currently, it is undergoing SLN testing in melanoma patients to evaluate the feasibility of using a combination of FDA-approved near-infrared fluorescent cRGDY-conjugated C dots and a handheld fluorescence imaging system to detect tumor-carrying lymph nodes in the intraoperative setting. A schematic illustration of SLN localization in the head and neck region is shown in Fig. 11.

In the design of lymphatic system-targeted nanomedicines, safety, efficacy, and potential for clinical translation are important factors to consider. Changing the preparation materials and methods of nanomedicines may lead to their clinical translation. A pure drug nanosystem based on supercritical technology that can be used for the pure drug nanonization of clinical fluorescent imaging agents [130, 131]. The constructed carrier-free indocyanine green nanoparticles (NanoICG) have smaller and more uniform sizes and better fluorescent performance compared to ICG molecules, without any molecular structure changes, and have demonstrated excellent imaging performance and effects in liver cancer surgery fluorescence navigation and lymph node imaging recognition. Importantly, this nanosystem can achieve stable loading of fluorescent dyes and targeted antibodies to prepare fluorescent probes with excellent targeting performance, providing an effective way for the precise identification of lymph nodes in clinical work [132].

In conclusion, while the progress in lymphatic drug delivery is substantial, clinical applications remain scarce. The future holds great potential for the refinement and expansion of this field, leveraging nanomedicine's unique advantages for lymphatic system delivery.

## Challenges and perspectives

The rapid advancement in molecular, biological, and genetic diagnostic technologies has sparked extensive exploration into diagnostic and imaging identification patterns within the lymphatic system, as well as investigations into its role in cancer initiation, progression, and metastasis. Among these, the development of nanomedicines offers a new direction for cancer intervention via the lymphatic pathway [133]. A significant advantage of nanocarrier drugs is their capacity to augment lymphatic drug absorption, enhance drug retention, and extend action time, building on the lymphatic system's inherent high permeability, retention effect, and structural and physiological functions [134]. For instance, the coupling of nanocarriers with antibodies or peptides enables the delivery of therapeutic drugs or dyes to specific lymph nodes, facilitating diagnostic and imaging identification guided excision, thus enriching the precision medicine repertoire [135]. In particular, the development of multifunctional nanoparticles may contribute significantly to the localization and diagnostic treatment of lymph nodes in various types of cancer. Additionally, nanocarrier formulations can deliver both imaging and therapeutic agents simultaneously, hence providing real-time monitoring of therapeutic effects during treatment through imaging.

Despite the innovative strides in nanotechnology resulting in the development of diverse lymphatic system nanocarriers, most designs remain at the stage of fundamental animal research. For further clinical progress, some key aspects should be considered for optimal clinical translation, such as: (1) Discerning if the combination or coupling of therapeutic agents, imaging agents, and carriers or ligands will change the pharmacokinetics and biodistribution of the carried drugs, and if it will cause new side effects; (2) Minimizing the toxicity of complex nanostructures to prevent or reduce the additional side effects of nanocarriers, such as cardiac, renal, and hepatic function damage, as well as damage to the nervous system caused by crossing the blood–brain barrier. These issues are crucial and have not yet been fully researched. Potential solutions may include altering the nanodrug preparation technology, optimizing the nanodrug preparation materials, and selecting suitable administration routes. For example, the pure drug nanotechnology could prepare drugs without using organic solvents or toxic solvents [136]. In addition, choosing clinical drugs or drug components as raw materials for preparing lymphatic system nanodrugs or selecting suitable routes of administration is expected to avoid serious side effects, simplify the process, and accelerate the clinical application process of nanodrugs. For example, several orally high lipophilic drugs based on modified or unmodified proteins or antibodies currently available on the market or in clinical trials, and non-enteric administration systems for biological preparations have shown the potential for lymphatic transmission, which can be explored or utilized.

Looking ahead, the advancement of material science and pharmaceutical science, particularly the construction of molecular conjugates with specific affinity for the lymphatic system, will further propel the development of lymph system-specific nanomedicines. The production of superior lymphatic system-targeted nanomedicines requires the improvement of nanomedicine preparation techniques and enhanced material science research. This includes: (1) Developing ligands targeting lymph node-specific biomarkers to increase nanoparticle retention in lymph nodes through active targeting; (2) Optimization of nanoparticle size, shape, and surface properties to improve passive targeting efficiency; (3) Designing lymphatic system microenvironment-specific responsive nanoparticle carriers to achieve targeted delivery to lymph nodes and even specific cells within the lymph nodes; 4) Advancing environmentally friendly and safe nanotechnology preparation techniques to increase the rate of clinical translation. These methods will facilitate the development of efficient and lymphatic system-targeted nanomedicines.

Furthermore, to promote the development of lymphatic system nanomedicines, there is a need to enhance our understanding of the structure, physiological functions, and lymphatic biology of the lymphatic system itself. Lymphatic acquisition is not only determined by size, but also by a series of transport and metabolic processes. Additionally, the occurrence and progression of cancer are interconnected with the status of lymphatic vessels and lymph nodes. The staging of cancer can affect the structure and function of the lymphatic system, and the physiological and treatment status of the lymphatic system can also impact the outcome of cancer. Limited understanding of tumor heterogeneity and lymphatic remodeling during tumor progression hinders the clinical translation of lymphocyte-targeted therapies. By modulating the immune response through the lymphatic system, reconstructing tumor-related microenvironments, and their integration, it may be possible to change the paradigm of cancer treatment and significantly improve patient survival rates in the foreseeable future [137]. Therefore, in the future, nanomedicines may focus on the overall disease progression process and the interactions between drugs, which could be a key area of research.

In summary, this review article summarizes various lymphatic targeting drug delivery methods based on strategies such as nanoparticle-based approaches, viral vectors, immune cells, and targeting ligands. These methods not only play important roles in cancer and lymph node metastasis treatment but also provide important references for the treatment of other diseases such as tuberculosis, HIV/AIDS, and influenza. For instance, in the treatment of tuberculosis, lipid nanoparticles or polymer nanoparticles can be prepared using nanotechnology, and their size, surface modification, and drug encapsulation can be adjusted to selectively accumulate in the lymph nodes after injection, thereby achieving improved therapeutic effects. In the treatment of HIV/AIDS, viral vectors can be used to deliver vaccine genes into lymph nodes, activating the immune response of the lymphatic system. This targeted immune activation can enhance immune protection against HIV, providing new strategies for the treatment of HIV/AIDS. Additionally, in the treatment of viral infections such as influenza, nanoparticles or viral vectors can be utilized to directly target and detect infected lymph nodes, enabling precise drug delivery and early detection of infections, thereby providing crucial information for treatment and control strategies.

## Data Availability

Not applicable.

## Abbreviations

FDA: The US Food and Drug Administration
EMA: European Medicines Agency
SCS: Subcapsular sinus
APCs: Antigen-presenting cells
DDAB: Dimethyl dioctadecyl ammonium bromide
TDB: Trehalose-6,6-dibehenate
TLR: Toll-like receptor
PolyIC: Polyinosinic-polycytidylic acid
T-MP: Curcumin-loaded porphyrin micelles
HSA: Human serum albumin
MSNs: Mesoporous silica nanoparticles
MSRs: Mesoporous silica rods
PEI: Polyethyleneimine
aMT-exos: A hybrid exosomes
AIDS: Acquired immunodeficiency syndrome
RES: Reticuloendothelial system
CRGDY: Cyclic arginine-glycine-aspartate peptide

## References

[1] Kase AM, Menke D, Tan W (2018). Breast cancer metastasis to the bladder: a literature review. BMJ Case Rep.

[2] Eckhardt BL, Cao Y, Redfern AD, Chi LH, Burrows AD, Roslan S, Sloan EK, Parker BS, Loi S, Ueno NT, Lau PKH, Latham B, Anderson RL (2020). Activation of canonical BMP4-SMAD7 signaling suppresses breast cancer metastasis. Cancer Res.

[3] Chen X, Wang W, Jiang Y, Qian X (2023). A dual-transformation with contrastive learning framework for lymph node metastasis prediction in pancreatic cancer. Med Image Anal.

[4] Ho AS, Kim S, Tighiouart M, Gudino C, Mita A, Scher KS, Laury A, Prasad R, Shiao SL, Ali N, Patio C, Mallen-St Clair J, Van Eyk JE, Zumsteg ZS (2018). Association of quantitative metastatic lymph node burden with survival in hypopharyngeal and laryngeal cancer. JAMA Oncol.

[5] Ye B, Fan D, Xiong W, Li M, Yuan J, Jiang Q, Zhao Y, Lin J, Liu J, Lv Y, Wang X, Li Z, Su J, Qiao Y (2021). Oncogenic enhancers drive esophageal squamous cell carcinogenesis and metastasis. Nat Commun.

[6] Li F, Nie W, Zhang F, Lu G, Lv C, Lv Y, Bao W, Zhang L, Wang S, Gao X, Wei W, Xie HY (2019). Engineering magnetosomes for high-performance cancer vaccination. ACS Cent Sci.

[7] Maeda H (2015). Toward a full understanding of the EPR effect in primary and metastatic tumors as well as issues related to its heterogeneity. Adv Drug Deliv Rev.

[8] Scott EA, Karabin NB, Augsornworawat P (2017). Overcoming immune dysregulation with immunoengineered nanobiomaterials. Annu Rev Biomed Eng.

[9] Zahin N, Anwar R, Tewari D, Kabir MT, Sajid A, Mathew B, Uddin MS, Aleya L, Abdel-Daim MM (2020). Nanoparticles and its biomedical applications in health and diseases: special focus on drug delivery. Environ Sci Pollut Res Int.

[10] Hoshyar N, Gray S, Han H, Bao G (2016). The effect of nanoparticle size on in vivo pharmacokinetics and cellular interaction. Nanomedicine.

[11] Zwicke GL, Mansoori GA, Jeffery CJ (2012). Utilizing the folate receptor for active targeting of cancer nanotherapeutics. Nano Rev.

[12] Lin N, Qiu J, Song J, Yu C, Fang Y, Wu W, Yang W, Wang Y (2021). Application of nano-carbon and titanium clip combined labeling in robot-assisted laparoscopic transverse colon cancer surgery. BMC Surg.

[13] Altundag K, Dede DS, Purnak T (2007). Albumin-bound paclitaxel (ABI-007; Abraxane) in the management of basal-like breast carcinoma. J Clin Pathol.

[14] Wang B, An J, Zhang H, Zhang S, Zhang H, Wang L, Zhang H, Zhang Z (2018). Personalized cancer immunotherapy via transporting endogenous tumor antigens to lymph nodes mediated by nano Fe3 O4. Small.

[15] Ryan GM, Kaminskas LM, Porter CJ (2014). Nano-chemotherapeutics: maximising lymphatic drug exposure to improve the treatment of lymph-metastatic cancers. J Control Release.

[16] Reddy ST, Rehor A, Schmoekel HG, Hubbell JA, Swartz MA (2006). In vivo targeting of dendritic cells in lymph nodes with poly (propylene sulfide) nanoparticles. J Control Release.

[17] Schudel A, Francis DM, Thomas SN (2019). Material design for lymph node drug delivery. Nat Rev Mater.

[18] Baluk P, Fuxe J, Hashizume H, Romano T, Lashnits E, Butz S, Vestweber D, Corada M, Molendini C, Dejana E, McDonald DM (2007). Functionally specialized junctions between endothelial cells of lymphatic vessels. J Exp Med.

[19] Oh HJ, Yang D, Oh HW, Jeon JG, Kim C, Ahn JY, Han SW, Kim CY (2020). Chronologic trends of cancer-related lymph node research in PubMed: informetrics analysis. Ann Surg Treat Res.

[20] Scallan JP, Zawieja SD, Castorena-Gonzalez JA, Davis MJ (2016). Lymphatic pumping: mechanics, mechanisms and malfunction. J Physiol.

[21] Gashev AA (2010). Basic mechanisms controlling lymph transport in the mesenteric lymphatic net. Ann N Y Acad Sci.

[22] Moore JE, Bertram CD (2018). Lymphatic system flows. Annu Rev Fluid Mech.

[23] Clement CC, Wang W, Dzieciatkowska M, Cortese M, Hansen KC, Becerra A, Thangaswamy S, Nizamutdinova I, Moon JY, Stern LJ, Gashev AA, Zawieja D, Santambrogio L (2018). Quantitative profiling of the lymph node clearance capacity. Sci Rep.

[24] Gerner MY, Torabi-Parizi P, Germain RN (2015). Strategically localized dendritic cells promote rapid T cell responses to lymph-borne particulate antigens. Immunity.

[25] Liu Y, Liu Y, Xu D, Zang J, Zheng X, Zhao Y, Li Y, He R, Ruan S, Dong H, Gu J, Yang Y, Cheng Q, Li Y (2022). Targeting the negative feedback of adenosine-A2AR metabolic pathway by a tailored nanoinhibitor for photothermal immunotherapy. Adv Sci.

[26] Jalkanen S, Salmi M (2020). Lymphatic endothelial cells of the lymph node. Nat Rev Immunol.

[27] Roozendaal R, Mebius RE, Kraal G (2008). The conduit system of the lymph node. Int Immunol.

[28] Palframan RT, Jung S, Cheng G, Weninger W, Luo Y, Dorf M, Littman DR, Rollins BJ, Zweerink H, Rot A, von Andrian UH (2001). Inflammatory chemokine transport and presentation in HEV: a remote control mechanism for monocyte recruitment to lymph nodes in inflamed tissues. J Exp Med.

[29] Schudel A, Chapman AP, Yau MK, Higginson CJ, Francis DM, Manspeaker MP, Avecilla ARC, Rohner NA, Finn MG, Thomas SN (2020). Programmable multistage drug delivery to lymph nodes. Nat Nanotechnol.

[30] Dukhin SS, Labib ME (2013). Convective diffusion of nanoparticles from the epithelial barrier toward regional lymph nodes. Adv Colloid Interface Sci.

[31] Ke X, Howard GP, Tang H, Cheng B, Saung MT, Santos JL, Mao HQ (2019). Physical and chemical profiles of nanoparticles for lymphatic targeting. Adv Drug Deliv Rev.

[32] Patravale VB, Prabhu RH, Bora CR (2017). Lymphatic delivery: concept, challenges and applications. Indian Drugs.

[33] Hawley AE, Davis SS, Illum L (1995). Targeting of colloids to lymph nodes: influence of lymphatic physiology and colloidal characteristics. Adv Drug Deliv Rev.

[34] Geng Y, Dalhaimer P, Cai S, Tsai R, Tewari M, Minko T, Discher DE (2007). Shape effects of filaments versus spherical particles in flow and drug delivery. Nat Nanotechnol.

[35] Ja C, Mitragotri S (2006). Role of target geometry in phagocytosis. Proc Natl Acad Sci USA.

[36] Montes-Casado M, Sanvicente A, Casarrubios L, Feito MJ, Rojo JM, Vallet-Regí M, Arcos D, Portolés P, Portolés MT (2020). An immunological approach to the biocompatibility of mesoporous SiO2-CaO nanospheres. Int J Mol Sci.

[37] Patel HM, Boodle KM, Vaughan-Jones R (1984). Assessment of the potential uses of liposomes for lymphoscintigraphy and lymphatic drug delivery. Failure of 99m-technetium marker to represent intact liposomes in lymph nodes. Biochim Biophys Acta.

[38] Punjabi MS, Naha A, Shetty D, Nayak UY (2021). Lymphatic drug transport and associated drug delivery technologies: a comprehensive review. Curr Pharm Des.

[39] Ding Y, Li Z, Jaklenec A, Hu Q (2021). Vaccine delivery systems toward lymph nodes. Adv Drug Deliv Rev.

[40] Chen Y, De Koker S, De Geest BG (2020). Engineering strategies for lymph node targeted immune activation. Acc Chem Res.

[41] Gracia G, Cao E, Feeney OM, Johnston APR, Porter CJH, Trevaskis NL (2020). High-density lipoprotein composition influences lymphatic transport after subcutaneous administration. Mol Pharm.

[42] He X, Wang J, Tang Y, Chiang ST, Han T, Chen Q, Qian C, Shen X, Li R, Ai X (2023). Recent advances of emerging spleen-targeting nanovaccines for immunotherapy. Adv Healthc Mater.

[43] Menon I, Bagwe P, Gomes KB, Bajaj L, Gala R, Uddin MN, D'Souza MJ, Zughaier SM (2021). Microneedles: a new generation vaccine delivery system. Micromachines.

[44] Trac N, Chung EJ (2021). Overcoming physiological barriers by nanoparticles for intravenous drug delivery to the lymph nodes. Exp Biol Med (Maywood).

[45] Furubayashi T, Inoue D, Kimura S, Tanaka A, Sakane T (2021). Evaluation of the pharmacokinetics of intranasal drug delivery for targeting cervical lymph nodes in rats. Pharmaceutics.

[46] Schudel A, Francis DM, Thomas SN (2019). Material design for lymph node drug delivery. Nat Rev Mater.

[47] Yoshida T, Kojima H, Sako K, Kondo H (2022). Drug delivery to the intestinal lymph by oral formulations. Pharm Dev Technol.

[48] Refaat H, Naguib YW, Elsayed MMA, Sarhan HAA, Alaaeldin E (2019). Modified spraying technique and response surface methodology for the preparation and optimization of propolis liposomes of enhanced anti-proliferative activity against human melanoma cell Line A375. Pharmaceutics.

[49] Bangham AD, Horne RW (1964). Negative staining of phospholipids and their structural modification by surface-active agents as observed in the electron microscope. J Mol Biol.

[50] Sun S, Sun S, Sun Y, Wang P, Zhang J, Du W, Wang S, Liang X (2019). Bubble-manipulated local drug release from a smart thermosensitive cerasome for dual-mode imaging guided tumor chemo-photothermal therapy. Theranostics.

[51] Reichmuth AM, Oberli MA, Jaklenec A, Langer R, Blankschtein D (2016). mRNA vaccine delivery using lipid nanoparticles. Ther Deliv.

[52] Jung HS, Neuman KC (2021). Surface Modification of fluorescent nanodiamonds for biological applications. Nanomaterials.

[53] Maeki M, Kimura N, Sato Y, Harashima H, Tokeshi M (2018). Advances in microfluidics for lipid nanoparticles and extracellular vesicles and applications in drug delivery systems. Adv Drug Deliv Rev.

[54] Milicic A, Kaur R, Reyes-Sandoval A, Tang CK, Honeycutt J, Perrie Y, Hill AV (2012). Small cationic DDA:TDB liposomes as protein vaccine adjuvants obviate the need for TLR agonists in inducing cellular and humoral responses. PLoS ONE.

[55] Chu Y, Qian L, Ke Y, Feng X, Chen X, Liu F, Yu L, Zhang L, Tao Y, Xu R, Wei J, Liu B, Liu Q (2022). Lymph node-targeted neoantigen nanovaccines potentiate anti-tumor immune responses of post-surgical melanoma. J Nanobiotechnol.

[56] Warashina S, Nakamura T, Sato Y, Fujiwara Y, Hyodo M, Hatakeyama H, Harashima H (2016). A lipid nanoparticle for the efficient delivery of siRNA to dendritic cells. J Control Release.

[57] Hanson MC, Crespo MP, Abraham W, Moynihan KD, Szeto GL, Chen SH, Melo MB, Mueller S, Irvine DJ (2015). Nanoparticulate STING agonists are potent lymph node-targeted vaccine adjuvants. J Clin Invest.

[58] Chen J, Ye Z, Huang C, Qiu M, Song D, Li Y, Xu Q (2022). Lipid nanoparticle-mediated lymph node-targeting delivery of mRNA cancer vaccine elicits robust CD8+ T cell response. Proc Natl Acad Sci USA.

[59] Phosphatidylserine lipid nanoparticles promote systemic RNA delivery to secondary lymphoid organs. Nano Lett. 2022; 22 (20): 8304–8311. 10.1021/acs.nanolett.2c03234 PMC12126148 36194390

[60] Trimaille T, Verrier B (2015). Micelle-based adjuvants for subunit vaccine delivery. Vaccines.

[61] Li C, Iqbal M, Jiang B, Wang Z, Kim J, Nanjundan AK, Whitten AE, Wood K, Yamauchi Y (2019). Pore-tuning to boost the electrocatalytic activity of polymeric micelle-templated mesoporous Pd nanoparticles. Chem Sci.

[62] Cui M, Jin M, Han M, Zang Y, Li C, Zhang D, Huang W, Gao Z, Yin X (2020). Improved antitumor outcomes for colon cancer using nanomicelles loaded with the novel antitumor agent LA67. Int J Nanomed.

[63] Li X, Dong Q, Yan Z, Lu W, Feng L, Xie C, Xie Z, Su B, Liu M (2015). MPEG-DSPE polymeric micelle for translymphatic chemotherapy of lymph node metastasis. Int J Pharm.

[64] Thol K, Pawlik P, McGranahan N (2022). Therapy sculpts the complex interplay between cancer and the immune system during tumour evolution. Genome Med.

[65] Ehser S, Chuang JJ, Kleist C, Sandra-Petrescu F, Iancu M, Wang D, Opelz G, Terness P (2008). Suppressive dendritic cells as a tool for controlling allograft rejection in organ transplantation: promises and difficulties. Hum Immunol.

[66] Jewell CM, López SC, Irvine DJ (2011). In situ engineering of the lymph node microenvironment via intranodal injection of adjuvant-releasing polymer particles. Proc Natl Acad Sci USA.

[67] Chida T, Miura Y, Cabral H, Nomoto T, Kataoka K, Nishiyama N (2018). Epirubicin-loaded polymeric micelles effectively treat axillary lymph nodes metastasis of breast cancer through selective accumulation and pH-triggered drug release. J Control Release.

[68] Cabral H, Makino J, Matsumoto Y, Mi P, Wu H, Nomoto T, Toh K, Yamada N, Higuchi Y, Konishi S, Kano MR, Nishihara H, Miura Y, Nishiyama N, Kataoka K (2015). Systemic targeting of lymph node metastasis through the blood vascular system by using size-controlled nanocarriers. ACS Nano.

[69] Feng HY, Yuan Y, Zhang Y, Liu HJ, Dong X, Yang SC, Liu XL, Lai X, Zhu MH, Wang J, Lu Q, Lin Q, Chen HZ, Lovell JF, Sun P, Fang C (2021). Targeted micellar phthalocyanine for lymph node metastasis homing and photothermal therapy in an orthotopic colorectal tumor model. Nanomicro Lett.

[70] Kumar A, Tan A, Wong J, Spagnoli JC, Lam J, Blevins BD, Thorne GNL, Ashkan K, Xie J, Liu H (2017). Nanotechnology for neuroscience: Promising approaches for diagnostics, therapeutics and brain activity mapping. Adv Funct Mater.

[71] Anraku Y, Kuwahara H, Fukusato Y, Mizoguchi A, Ishii T, Nitta K, Matsumoto Y, Toh K, Miyata K, Uchida S, Nishina K, Osada K, Itaka K, Nishiyama N, Mizusawa H, Yamasoba T, Yokota T, Kataoka K (2017). Glycaemic control boosts glucosylated nanocarrier crossing the BBB into the brain. Nat Commun.

[72] Xu C, Feng Q, Yang H, Wang G, Huang L, Bai Q, Zhang C, Wang Y, Chen Y, Cheng Q, Chen M, Han Y, Yu Z, Lesniak MS, Cheng Y (2018). A Light-triggered mesenchymal stem cell delivery system for photoacoustic imaging and chemo-photothermal therapy of triple negative breast cancer. Adv Sci.

[73] Liu Y, Wang Z, Yu F, Li M, Zhu H, Wang K, Meng M, Zhao W (2021). The adjuvant of α-galactosylceramide presented by gold nanoparticles enhances antitumor immune responses of MUC1 antigen-based tumor vaccines. Int J Nanomedicine.

[74] Mottas I, Bekdemir A, Cereghetti A, Spagnuolo L, Yang YS, Müller M, Irvine DJ, Stellacci F, Bourquin C (2019). Amphiphilic nanoparticle delivery enhances the anticancer efficacy of a TLR7 ligand via local immune activation. Biomaterials.

[75] Oladipo AO, Oluwafemi OS, Songca SP, Sukhbaatar A, Mori S, Okajima J, Komiya A, Maruyama S, Kodama T (2017). A novel treatment for metastatic lymph nodes using lymphatic delivery and photothermal therapy. Sci Rep.

[76] Dadfar SM, Roemhild K, Drude NI, von Stillfried S, Knüchel R, Kiessling F, Lammers T (2019). Iron oxide nanoparticles: diagnostic, therapeutic and theranostic applications. Adv Drug Deliv Rev.

[77] Pourmadadi M, Rahmani E, Shamsabadipour A, Mahtabian S, Ahmadi M, Rahdar A, Díez-Pascual AM (2022). Role of iron oxide (Fe2O3) nanocomposites in advanced biomedical applications: a state-of-the-art review. Nanomaterials.

[78] Kjellman P, in 't Zandt R, Fredriksson S, Strand SE. 2014. Optimizing retention of multimodal imaging nanostructures in sentinel lymph nodes by nanoscale size tailoring. Nanomedicine. 2014;10: 1089–95. 10.1016/j.nano.2014.01.007 24502988

[79] Zaloga J, Janko C, Nowak J, Matuszak J, Knaup S, Eberbeck D, Tietze R, Unterweger H, Friedrich RP, Duerr S, Heimke-Brinck R, Baum E, Cicha I, Dörje F, Odenbach S, Lyer S, Lee G, Alexiou C (2014). Development of a lauric acid/albumin hybrid iron oxide nanoparticle system with improved biocompatibility. Int J Nanomedicine.

[80] Zou Y, Liu P, Liu CH, Zhi XT (2015). Doxorubicin-loaded mesoporous magnetic nanoparticles to induce apoptosis in breast cancer cells. Biomed Pharmacother.

[81] Quinto CA, Mohindra P, Tong S, Bao G (2015). Multifunctional superparamagnetic iron oxide nanoparticles for combined chemotherapy and hyperthermia cancer treatment. Nanoscale.

[82] Li AW, Sobral MC, Badrinath S, Choi Y, Graveline A, Stafford AG, Weaver JC, Dellacherie MO, Shih TY, Ali OA, Kim J, Wucherpfennig KW, Mooney DJ (2018). A facile approach to enhance antigen response for personalized cancer vaccination. Nat Mater.

[83] Lu Y, Yang Y, Gu Z, Zhang J, Song H, Xiang G, Yu C (2018). Glutathione-depletion mesoporous organosilica nanoparticles as a self-adjuvant and Co-delivery platform for enhanced cancer immunotherapy. Biomaterials.

[84] Khakpour E, Salehi S, Naghib SM, Ghorbanzadeh S, Zhang W (2023). Graphene-based nanomaterials for stimuli-sensitive controlled delivery of therapeutic molecules. Front Bioeng Biotechnol.

[85] Yang F, Jin C, Yang D, Jiang Y, Li J, Di Y, Hu J, Wang C, Ni Q, Fu D (2011). Magnetic functionalised carbon nanotubes as drug vehicles for cancer lymph node metastasis treatment. Eur J Cancer.

[86] Wang J, Lu T, Yang M, Sun D, Xia Y, Wang T (2019). Hydrogel 3D printing with the capacitor edge effect. Sci Adv.

[87] Chen W, Chen H, Zheng D, Zhang H, Deng L, Cui W, Zhang Y, Santos HA, Shen H (2019). Gene-hydrogel microenvironment regulates extracellular matrix metabolism balance in nucleus pulposus. Adv Sci.

[88] Deng W, Yan Y, Zhuang P, Liu X, Tian K, Huang W, Li C (2022). Synthesis of nanocapsules blended polymeric hydrogel loaded with bupivacaine drug delivery system for local anesthetics and pain management. Drug Deliv.

[89] Zhuang X, Wu T, Zhao Y, Hu X, Bao Y, Guo Y, Song Q, Li G, Tan S, Zhang Z (2016). Lipid-enveloped zinc phosphate hybrid nanoparticles for codelivery of H-2K(b) and H-2D(b)-restricted antigenic peptides and monophosphoryl lipid A to induce antitumor immunity against melanoma. J Control Release.

[90] Nuhn L, Vanparijs N, De Beuckelaer A, Lybaert L, Verstraete G, Deswarte K, Lienenklaus S, Shukla NM, Salyer AC, Lambrecht BN, Grooten J, David SA, De Koker S, De Geest BG (2016). pH-degradable imidazoquinoline-ligated nanogels for lymph node-focused immune activation. Proc Natl Acad Sci USA.

[91] De Koker S, Cui J, Vanparijs N, Albertazzi L, Grooten J, Caruso F, De Geest BG (2016). Engineering polymer hydrogel nanoparticles for lymph node-targeted delivery. Angew Chem Int Ed Engl.

[92] Urimi D, Hellsing M, Mahmoudi N, Söderberg C, Widenbring R, Gedda L, Edwards K, Loftsson T, Schipper N (2022). Structural characterization study of a lipid nanocapsule formulation intended for drug delivery applications using small-angle scattering techniques. Mol Pharm.

[93] Shafiq M, Anjum S, Hano C, Anjum I, Abbasi BH (2020). An overview of the applications of nanomaterials and nanodevices in the food industry. Foods.

[94] Vicente S, Goins BA, Sanchez A, Alonso MJ, Phillips WT (2014). Biodistribution and lymph node retention of polysaccharide-based immunostimulating nanocapsules. Vaccine.

[95] Li AV, Moon JJ, Abraham W, Suh H, Elkhader J, Seidman MA, Yen M, Im EJ, Foley MH, Barouch DH, Irvine DJ (2013). Generation of effector memory T cell-based mucosal and systemic immunity with pulmonary nanoparticle vaccination. Sci Transl Med.

[96] Nawaz M, Yusuf N, Habib S, Shakoor RA, Ubaid F, Ahmad Z, Kahraman R, Mansour S, Gao W (2019). Development and properties of polymeric nanocomposite coatings. Polymers.

[97] Sato Y, Hashiba K, Sasaki K, Maeki M, Tokeshi M, Harashima H (2019). Understanding structure-activity relationships of pH-sensitive cationic lipids facilitates the rational identification of promising lipid nanoparticles for delivering siRNAs in vivo. J Control Release.

[98] Gao W, Fang RH, Thamphiwatana S, Luk BT, Li J, Angsantikul P, Zhang Q, Hu CM, Zhang L (2015). Modulating antibacterial immunity via bacterial membrane-coated nanoparticles. Nano Lett.

[99] Hu CM, Fang RH, Wang KC, Luk BT, Thamphiwatana S, Dehaini D, Nguyen P, Angsantikul P, Wen CH, Kroll AV, Carpenter C, Ramesh M, Qu V, Patel SH, Zhu J, Shi W, Hofman FM, Chen TC, Gao W, Zhang K, Chien S, Zhang L (2015). Nanoparticle biointerfacing by platelet membrane cloaking. Nature.

[100] Liu C, Liu X, Xiang X, Pang X, Chen S, Zhang Y, Ren E, Zhang L, Liu X, Lv P, Wang X, Luo W, Xia N, Chen X, Liu G (2022). A nanovaccine for antigen self-presentation and immunosuppression reversal as a personalized cancer immunotherapy strategy. Nat Nanotechnol.

[101] Wang S, Li F, Ye T, Wang J, Lyu C, Qing S, Ding Z, Gao X, Jia R, Yu D, Ren J, Wei W, Ma G (2021). Macrophage-tumor chimeric exosomes accumulate in lymph node and tumor to activate the immune response and the tumor microenvironment. Sci Transl Med.

[102] Hong D, Zhang L, Xu K, Wan X, Guo Y (2021). Prognostic value of pre-treatment CT radiomics and clinical factors for the overall survival of advanced (IIIB-IV) lung adenocarcinoma patients. Front Oncol.

[103] Chiechio RM, Ducarre S, Marets C, Dupont A, Even-Hernandez P, Pinson X, Dutertre S, Artzner F, Musumeci P, Ravel C, Faro MJL, Marchi V (2022). Encapsulation of luminescent gold nanoclusters into synthetic vesicles. Nanomaterials.

[104] Yoon HY, Chang IH, Goo YT, Kim CH, Kang TH, Kim SY, Lee SJ, Song SH, Whang YM, Choi YW (2019). Intravesical delivery of rapamycin via folate-modified liposomes dispersed in thermo-reversible hydrogel. Int J Nanomedicine.

[105] Osborne MP, Richardson VJ, Jeyasingh K, Ryman BE (1979). Radionuclide-labelled liposomes–a new lymph node imaging agent. Int J Nucl Med Biol.

[106] Phillips WT, Klipper R, Goins B (2000). Novel method of greatly enhanced delivery of liposomes to lymph nodes. J Pharmacol Exp Ther.

[107] Phillips WWT, Klipper R, Goins B (2001). Use of (99m)Tc-labeled liposomes encapsulating blue dye for identification of the sentinel lymph node. J Nucl Med.

[108] Yuan B, Zhao S, Hu P, Cui J, Niu QJ (2020). Asymmetric polyamide nanofilms with highly ordered nanovoids for water purification. Nat Commun.

[109] Esfand R, Tomalia DA (2001). Poly(amidoamine) (PAMAM) dendrimers: from biomimicry to drug delivery and biomedical applications. Drug Discov Today.

[110] Talanov VS, Regino CA, Kobayashi H, Bernardo M, Choyke PL, Brechbiel MW (2006). Dendrimer-based nanoprobe for dual modality magnetic resonance and fluorescence imaging. Nano Lett.

[111] Kobayashi H, Kawamoto S, Sakai Y, Choyke PL, Star RA, Brechbiel MW, Sato N, Tagaya Y, Morris JC, Waldmann TA (2004). Lymphatic drainage imaging of breast cancer in mice by micro-magnetic resonance lymphangiography using a nano-size paramagnetic contrast agent. J Natl Cancer Inst.

[112] Niki Y, Ogawa M, Makiura R, Magata Y, Kojima C (2015). Optimization of dendrimer structure for sentinel lymph node imaging: Effects of generation and terminal group. Nanomedicine.

[113] Yakunin S, Chaaban J, Benin BM, Cherniukh I, Bernasconi C, Landuyt A, Shynkarenko Y, Bolat S, Hofer C, Romanyuk YE, Cattaneo S, Pokutnyi SI, Schaller RD, Bodnarchuk MI, Poulikakos D, Kovalenko MV (2021). Radiative lifetime-encoded unicolour security tags using perovskite nanocrystals. Nat Commun.

[114] Han SJ, Rathinaraj P, Park SY, Kim YK, Lee JH, Kang IK, Moon JS, Winiarz JG (2014). Specific intracellular uptake of herceptin-conjugated CdSe/ZnS quantum dots into breast cancer cells. Biomed Res Int.

[115] Kim S, Lim YT, Soltesz EG, De Grand AM, Lee J, Nakayama A, Parker JA, Mihaljevic T, Laurence RG, Dor DM, Cohn LH, Bawendi MG, Frangioni JV (2004). Near-infrared fluorescent type II quantum dots for sentinel lymph node mapping. Nat Biotechnol.

[116] Kim SW, Zimmer JP, Ohnishi S, Tracy JB, Frangioni JV, Bawendi MG (2005). Engineering InAs(x)P(1–x)/InP/ZnSe III-V alloyed core/shell quantum dots for the near-infrared. J Am Chem Soc.

[117] Dai T, Zhou S, Yin C, Li S, Cao W, Liu W, Sun K, Dou H, Cao Y, Zhou G (2014). Dextran-based fluorescent nanoprobes for sentinel lymph node mapping. Biomaterials.

[118] Hultborn KA, Larsson LG, Raghult I (1955). The lymph drainage from the breast to the axillary and parasternal lymph nodes, studied with the aid of colloidal Au198. Acta radiol.

[119] Zhou Y, Chakraborty S, Liu S (2011). Radiolabeled cyclic RGD peptides as radiotracers for imaging tumors and thrombosis by SPECT. Theranostics.

[120] Wilhelm AJ, Mijnhout GS, Franssen EJ (1999). Radiopharmaceuticals in sentinel lymph-node detection—an overview. Eur J Nucl Med.

[121] Xie F, Zhang D, Cheng L, Yu L, Yang L, Tong F, Liu H, Wang S, Wang S (2015). Intradermal microbubbles and contrast-enhanced ultrasound (CEUS) is a feasible approach for sentinel lymph node identification in early-stage breast cancer. World J Surg Oncol.

[122] Montoya Mira J, Wu L, Sabuncu S, Sapre A, Civitci F, Ibsen S, Esener S, Yildirim A (2020). Gas-stabilizing sub-100 nm mesoporous silica nanoparticles for ultrasound theranostics. ACS Omega.

[123] Nie Z, Luo N, Liu J, Zeng X, Zhang Y, Su D (2020). Multi-mode biodegradable tumour-microenvironment sensitive nanoparticles for targeted breast cancer imaging. Nanoscale Res Lett.

[124] Stride E, Saffari N (2004). The potential for thermal damage posed by microbubble ultrasound contrast agents. Ultrasonics.

[125] Ma JJ, Zhang DB, Zhang WF, Wang X (2020). Application of nanocarbon in breast approach endoscopic thyroidectomy thyroid cancer surgery. J Laparoendosc Adv Surg Tech A.

[126] Wang R, Mo S, Liu Q, Zhang W, Zhang Z, He Y, Cai G, Li X (2020). The safety and effectiveness of carbon nanoparticles suspension in tracking lymph node metastases of colorectal cancer: a prospective randomized controlled trial. Jpn J Clin Oncol.

[127] Schilham MGM, Zamecnik P, Privé BM, Israël B, Rijpkema M, Scheenen T, Barentsz JO, Nagarajah J, Gotthardt M (2021). Head-to-head comparison of 68Ga-prostate-specific membrane antigen PET/CT and ferumoxtran-10-enhanced MRI for the diagnosis of lymph node metastases in prostate cancer patients. J Nucl Med.

[128] Wallace AM, Hoh CK, Ellner SJ, Darrah DD, Schulteis G, Vera DR (2007). Lymphoseek: a molecular imaging agent for melanoma sentinel lymph node mapping. Ann Surg Oncol.

[129] Bradbury MS, Pauliah M, Zanzonico P, Wiesner U, Patel S (2016). Intraoperative mapping of sentinel lymph node metastases using a clinically translated ultrasmall silica nanoparticle. Wiley Interdiscip Rev Nanomed Nanobiotechnol.

[130] He P, Ren E, Chen B, Chen H, Cheng H, Gao X, Liang X, Liu H, Li J, Li B, Chen A, Chu C, Chen X, Mao J, Zhang Y, Liu G (2022). A super-stable homogeneous lipiodol-hydrophilic chemodrug formulation for treatment of hepatocellular carcinoma. Theranostics.

[131] He P, Xiong Y, Ye J, Chen B, Cheng H, Liu H, Zheng Y, Chu C, Mao J, Chen A, Zhang Y, Li J, Tian J, Liu G (2022). A clinical trial of super-stable homogeneous lipiodol-nanoICG formulation-guided precise fluorescent laparoscopic hepatocellular carcinoma resection. J Nanobiotechnology.

[132] Zhang Y, Cheng H, Chen H, Xu P, Ren E, Jiang Y, Li D, Gao X, Zheng Y, He P, Lin H, Chen B, Lin G, Chen A, Chu C, Mao J, Liu G (2022). A pure nanoICG-based homogeneous lipiodol formulation: toward precise surgical navigation of primary liver cancer after long-term transcatheter arterial embolization. Eur J Nucl Med Mol Imaging.

[133] Naz S, Shamoon M, Wang R, Zhang L, Zhou J, Chen J (2019). Advances in therapeutic implications of inorganic drug delivery nano-platforms for cancer. Int J Mol Sci.

[134] Eid HM, Ali AA, Ali AMA, Eissa EM, Hassan RM, Abo El-Ela FI, Hassan AH (2022). Potential use of tailored citicoline chitosan-coated liposomes for effective wound healing in diabetic rat model. Int J Nanomedicine.

[135] Lee J, Kang S, Park H, Sun JG, Kim EC, Shim G (2023). Nanoparticles for lymph node-directed delivery. Pharmaceutics.

[136] Cheng H, Yang X, Liu G (2020). Superstable homogeneous iodinated formulation technology: revolutionizing transcatheter arterial chemoembolization. Sci Bull.

[137] Peng X, Wang J, Zhou F, Liu Q, Zhang Z (2021). Nanoparticle-based approaches to target the lymphatic system for antitumor treatment. Cell Mol Life Sci.

